# Global, regional, and national analyses of the burden of colorectal cancer attributable to diet low in milk from 1990 to 2019: longitudinal observational study

**DOI:** 10.3389/fnut.2024.1431962

**Published:** 2024-07-22

**Authors:** Xingxing Zhang, Xinru Zhang, Ruihua Li, Meiyan Lin, Tongyin Ou, Hu Zhou, Zhiming Chen, Li Zhen

**Affiliations:** ^1^Department of General Surgery, Nanfang Hospital, Southern Medical University, Guangzhou, China; ^2^School of Nursing, Southern Medical University, Guangzhou, China; ^3^Department of Thoracic Surgery, Nanfang Hospital, Southern Medical University, Guangzhou, China

**Keywords:** colorectal cancer, diet low in milk, Global Burden of Disease, disability-adjusted life year, GBD 2019

## Abstract

**Background:**

Globally, diet low in milk is the third greatest risk factor for colorectal cancer (CRC). However, there has been a lack of detailed worldwide analysis of the burden and trends of CRC attributable to diet low in milk.

**Objective:**

We aim to assess the spatiotemporal trends of CRC-related mortality and disability-adjusted life-years (DALYs) attributable to diet low in milk at the global, regional, and national levels from 1990 to 2019.

**Methods:**

Data of mortality, DALYs, age-standardized mortality rate (ASMR), and age-standardized DALY rate (ASDR) of CRC attributable to diet low in milk were extracted from the Global Burden of Disease (GBD) 2019 study. The burden of CRC attributable to diet low in milk was estimated using the ASMR and ASDR, while accounting for sex, age, country, and socio-demographic index (SDI). From 1990 to 2019, the estimated annual percentage change (EAPC) was calculated to clarify the temporal trends in the ASMR and ASDR attributable to diet low in milk.

**Results:**

In 2019, there were 166,456 (95% UI = 107,221–226,027) deaths and 3,799,297 (95% UI = 2,457,768–5,124,453) DALYs attributable to diet low in milk, accounting for 15.3 and 15.6% of CRC-related deaths and DALYs in 2019. CRC-related deaths and DALYs attributed to diet low in milk increased by 130.5 and 115.4%, from 1990 to 2019. The burden of CRC attributable to diet low in milk varied notably among regions and nations. High-middle SDI regions had the highest ASDR and ASMR of CRC linked to diet low in milk, while there was a slight downward trend high SDI regions. Among geographical regions, East Asia had the highest number of CRC-related deaths and DALYs attributable to diet low in milk. Notably, the burden of CRC was highest in males and the elderly. With coefficients of −0.36 and −0.36, the EAPC in ASMR and ASDR was significantly inversely correlated with the Human Development Index in 2019.

**Conclusion:**

Globally, the number of CRC deaths attributable to diet low in milk has continued to increase over the last 30 years. Therefore, government and authorities should conduct education campaigns to encourage individuals to increase daily milk intake.

## 1 Introduction

As the second most common malignancy and the third most common cause of cancer-related death globally, colorectal cancer (CRC) accounted for more than 2.1 million (7%) of all new cancer cases and 1.0 million (11%) of all cancer-related deaths worldwide in 2019 (1). There are considerable geographic variations in the burden of CRC, which is strongly associated with socioeconomic status (2). Traditionally, the incidence and mortality rates of CRC are highest in Europe, Oceania, and North America (3, 4). However, the incidence rates are rising in high-middle socio-demographic index (SDI) regions, notably in East Asia, Eastern Europe, Asia, and South America, as a result of economic improvements and shifts in dietary patterns and lifestyles (5, 6). Consequently, CRC remains a significant economic and medical challenge globally.

Multiple risk factors would promote the malignant development of CRC, such as genetic, age, environmental, lifestyle (dietary habits and activities) and metabolic risks (7). However, the exact cause of CRC is still unknown, and these risk factors may lead to potential confounders that could lead to spurious relationships in the observed data. In the GBD study, the three highest attributable proportions of risk factors of CRC in 2019 were diet low in whole grain diet (15.8%), diet low in milk (15.3%), and smoking (12.9%) (8). The risk attributable to diet low in milk have exceeded the risk attributable to diet high in processed meat. Meanwhile, the proportion of CRC attributable risk attributable to diet high in processed meat diet decreased (9). Milk, as a cheap and commonly consumed food worldwide, can provides a variety of macronutrients, micronutrients, and bioactive components that are crucial to growth and development (10). In addition, milk conveys various potential health advantages, including anti-cancer, anti-inflammatory, antioxidant, anti-fat, anti-hypertension, anti-hyperglycemia, and anti-osteoporosis activities (11). A recent meta-analysis of 15 cohort studies involving 11,733 individuals found that higher consumption of total dairy products and milk may be associated with a decreased risk of CRC (12). Moreover, the American Institute for Cancer Research and the World Cancer Research Fund found that drinking milk may reduce the incidence of CRC (13). However, the majority of the population does not reach the recommended daily milk intake, and there is a lack of updated epidemiological research analyzing colorectal cancer due to low dietary milk levels from a global perspective. Therefore, it is necessary to systematically explore the burden of colorectal cancer attributable to diet low in milk.

The Global Burden of Disease (GBD) 2019 study provides comprehensive and up-to-date data of 369 diseases and injuries, as well as 87 risk factors, from more than 204 countries and territories worldwide (14). Several recent studies have utilized the GBD database to investigate the global, regional, and national burdens of CRC and identify associated risk factors. The results of these studies revealed that the primary risk factors for disability-adjusted life years (DALYs) associated with CRC across all countries and regions were diet low in milk, smoking, low calcium diets, and alcohol intake (7–9). A prior study (15) was conducted to analyze the burden and trend of CRC attributable to diet low in milk in China from 1990 to 2017, but this investigation only focused on a single country. To the best of our knowledge, there has not yet been any detailed worldwide analysis of the burden and trends of CRC attributable to diet low in milk.

Therefore, the aim of this comprehensive review was to quantified the global, regional, and national disease burden associated with CRC attributable to a low milk diet in reference to the most recent data from the GBD 2019 study. In addition, potential correlations among the human development index (HDI), sociodemographic index (SDI), and CRC burden were assessed. Moreover, the estimated annual percentage change (EAPC) was calculated in order to quantify the trend of the age-standardized rate (ASR) over time. The results of this study will help to better understand the impact of diet low in milk in order to decrease the incidence and burden of CRC.

## 2 Materials and methods

### 2.1 Data source

All data were derived from the GBD 2019 study, which was a cooperative international research project to estimate the burden of 286 causes of death, 369 diseases and injuries, and 87 risk factors in 204 countries or territories, 21 regions, and 5 SDI regions from 1990 to 2019 (16). The GBD 2019 research team collected raw data from civil registration, vital statistics, hospital records, and household surveys in each country, providing reliable estimates about the burden of colorectal cancer. DisMod-MR version 2.1 was used to adjust for bias in the raw data to provide internally consistent estimates of prevalence by age, gender, location, and year (17). With the use of the Global Health Data Exchange website,^1^ the number of CRC deaths and DALYs attributable to diet low in milk, as well as additional age-standardized mortality rates (ASMRs) and age-standardized DALY rates (ASDRs) of 204 countries and territories between January 1, 1990 and December 31, 2019 were obtained from the GBD 2019 study and stratified by sex, age, GBD region, and SDI quintile. The detailed methods for data input, mortality estimation, and modelling of the GBD 2019 study were obtained from earlier published articles (18). The searched terms included “colorectal cancer,” “diet low in milk,” “death” and “DALYs,” in addition to the years “1990–2010,” “1990–2019,” and “2010–2019,” as well as the metrics “number,” “percent,” and “rate.” The HDI values were retrieved from the United Nations Development Programme database.^2^

### 2.2 Definitions

The GBD 2019 study defined diet low in milk as an average daily intake of less than 360–500 g of whole, skim, and semiskim milk, excluding soy milk and other plant-based products (7). DALY is the sum of all healthy life-years lost between the onset of disease and mortality. The ASR was calculated based on age groups of the standard population. Because the total population mortality and DALY rate are influenced not only by the level of mortality and DALY rate of each age group, as well as the age composition of the population, the ASR provides the ability to eliminate the effect of the age composition of the population to enable more accurate comparisons of total mortality and DALY rates of different regions and time periods.

In addition, the correlation between the burden of disease and the SDI was analyzed. The SDI is a composite indicator of the average *per capita* income, fertility, and educational level of each country and region. The 204 countries and regions were divided into five categories based on the SDI: low (<0.45), low-middle (≥0.45 and <0.61), middle (≥0.61 and <0.69), high-middle (≥0.69 and <0.80), and high (≥0.80).

### 2.3 Statistical analysis

The burden of CRC attributable to diet low in milk was assessed by SDI, region, country, sex, and age group based on the number of deaths, DALYs, ASDR, and ASMR. The following formula was used to calculate the ASR:


$$ASR=\frac{\sum_{i=1}^{A}a_{i}w_{i}}{\sum_{i=1}^{A}w_{i}}\times 100,000\text{,}$$

where *a_i_* is the age-specific rate in the *i*th age group, *w* is the number of people (or the weight) in the respective *i*th age group from the chosen standard population, and *A* is the number of age groups. All rates are provided as per 100,000 persons in order to avoid effects due to the age composition of the populations. Rather than using the global population size in 2019, the weight was based on the global standard population (GBD 2019 study). Moreover, linear regression was used to calculate the EAPC, as linear regression research predicts the value of unknown data by using another relevant known data value. EAPC is approximately equal to the annual change over a specified time period, to evaluate trends in the ASR. The regression model used to assess trends in the ASR was *y* = *α + βx + ε*, where *x* is the calendar year, *y* is the ln(ASMR or ASDR), *ε* is the error term, and *β* is the positive or negative trend of the ASR. The exact calculation was EAPC = 100 (exp(*β*) − 1). The 95% confidence interval (CI) was obtained from the linear regression model. If the EAPC and 95% CI were >0, the ASR was considered an increasing trend or <0 as a decreasing trend. Otherwise, the ASR was considered stable. The 204 countries or territories were divided into four groups based on temporal trends using hierarchical cluster analysis: remained stable, minor increase, significant increase, or decrease. Finally, to identify variables influencing the EAPC, Pearson’s correlation analysis was performed to analyze the relationship between the EAPCs and ASR in 1990, as well as the relationship between the EAPCs and HDI in 2019. The reason for choosing Pearson’s correlation analysis is that it can effectively measure linear correlation in continuous data. All statistical analyses were performed using *R* software version 4.2.3.^3^ A probability (*p*) value <0.05 was considered statistically significant.

## 3 Results

### 3.1 Global trends of CRC attributable to diet low in milk

**Figure 1:**
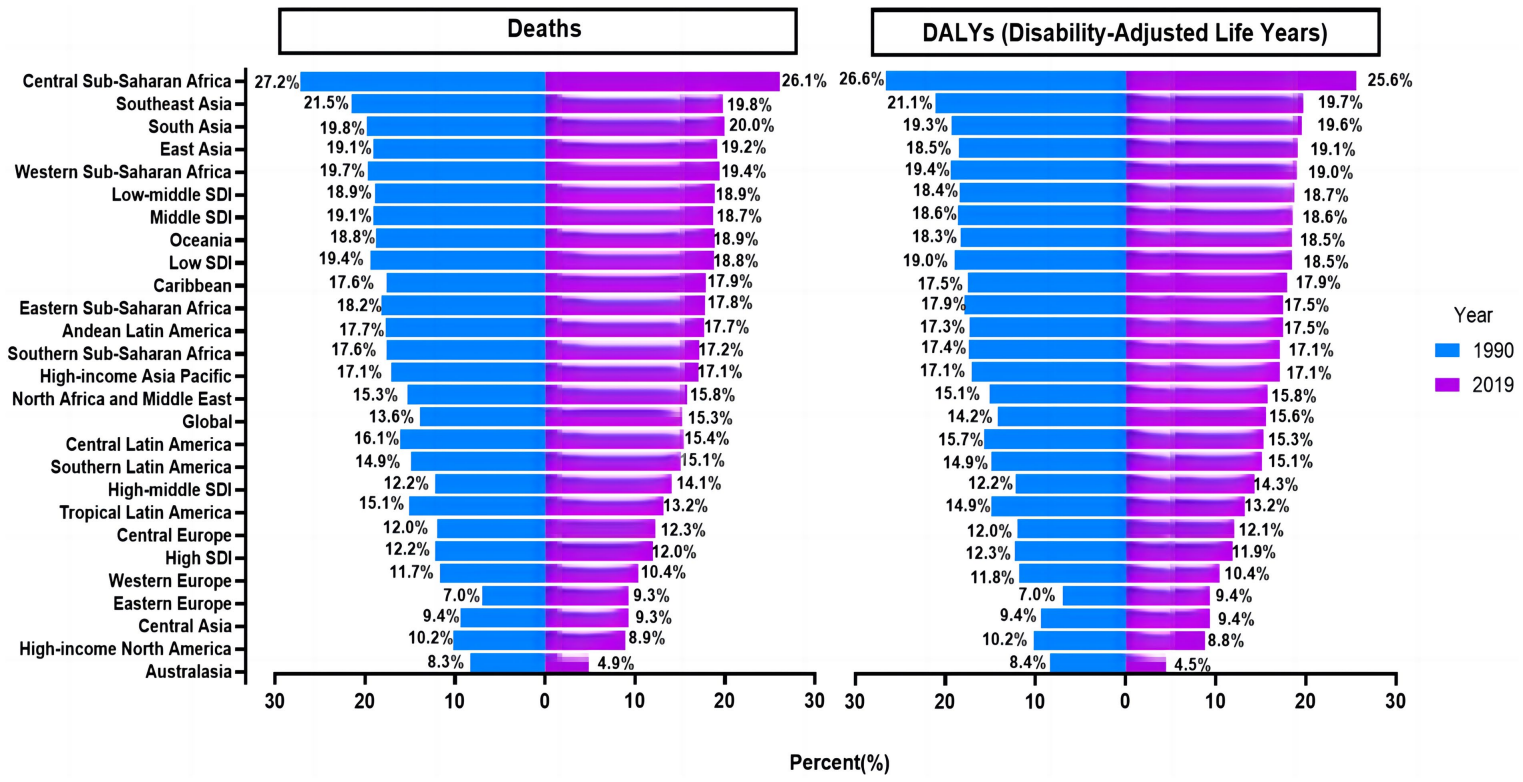
Proportion of colorectal cancer deaths and DALYs attributable to diet low in milk globally and in 26 GBD regions in 1990 and 2019. DALYs, disability-adjusted life-years; GBD, Global Burden of Disease Study.

**Figure 2:**
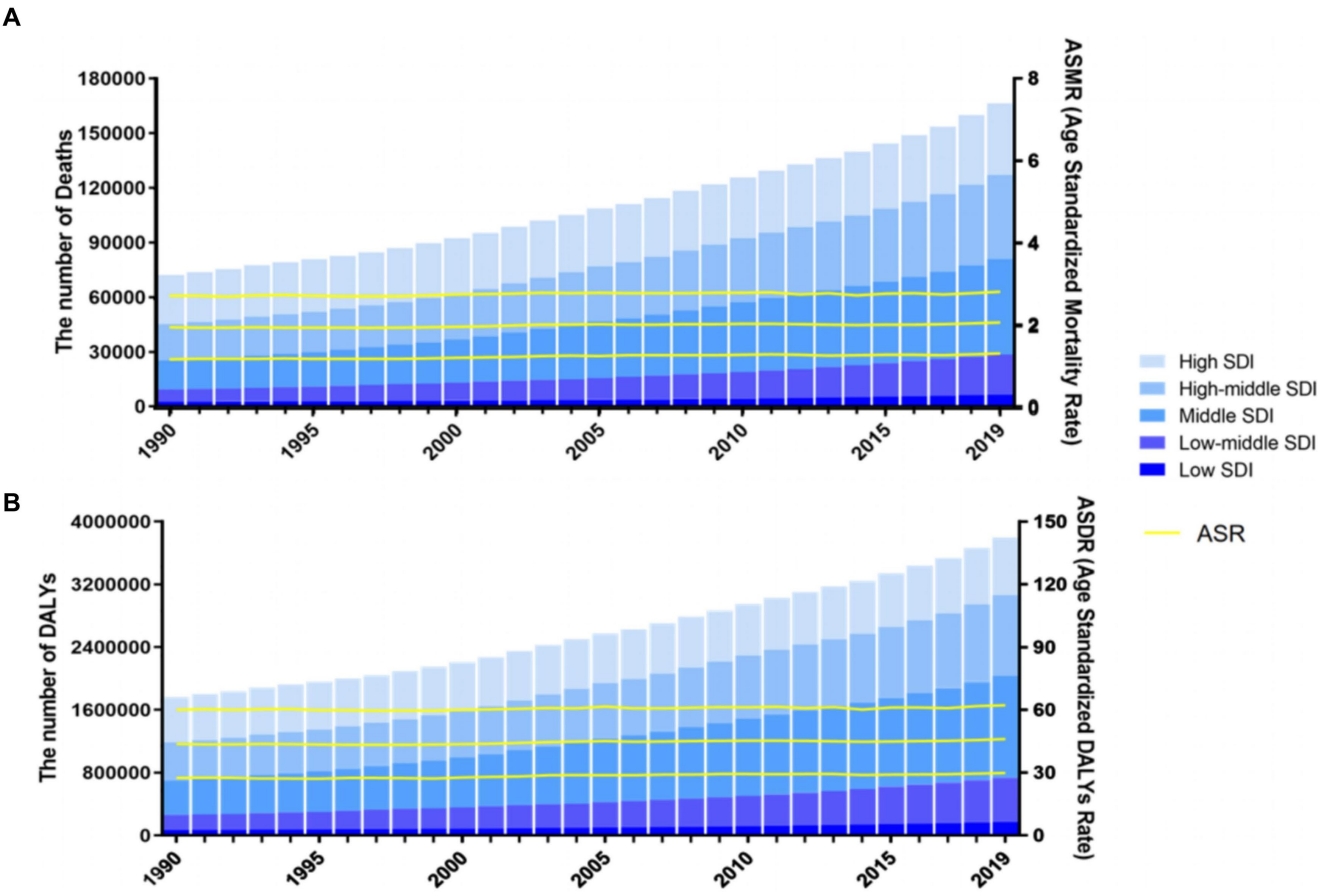
Number and rate of colorectal cancer deaths **(A)** and DALYS **(B)** attributable to diet low in milk from 1990 to 2019 by SDI level. The bars represent the number of colorectal cancer deaths **(A)** and DALYS **(B)** attributable to diet low in milk from 1990 to 2019 colored by SDI level. The line represents the mean ASMR **(A)** and ASDR **(B)** (per 100,000) attributable to diet low in milk at the global level. The shaded area represents the 95% UI for the mean rate. ASMR, age-standardized mortality rate; ASDR, age-standardized DALY rate; DALYs, disability-adjusted life-years; SDI, socio-demographic index; UI, uncertainty interval.

In 2019, the global number of CRC-related deaths and DALYs attributable to diet low in milk were estimated at 166,456 [95% uncertainty interval (UI) = 107,221–226,027] and 3,799,297 (95% UI = 2,457,768–5,124,453), representing 15.3 and 15.6% of all CRC-related deaths and DALYs, respectively (Figure 1). Between 1990 and 2019, the number of global deaths from CRC associated with diet low in milk increased by 130.5%, from 72,199 (95% UI = 44,384–99,966) to 166,456 (95% UI = 107,221–226,027), respectively. Similarly, the number of DALYs increased by 115.4%, from 1,764,172 (95% UI = 1,115,033–2,420,485) to 3,799,297 (95% UI = 2,457,768–5,124,453), respectively. In 2019, the ASDR and ASMR were 46.09 (95% UI = 29.83–62.18) and 2.09 (95% UI = 1.34–2.84) per 100,000, respectively. Both the global CRC-associated ASMR and ASDR due to diet low milk had modestly increased, with EAPCs of 0.19% [95% Confidence Interval (CI) = 0.16–0.23] and 0.19% (95% CI = 0.16–0.22), respectively (Table 1; Figure 2).

**Table 1:** Global burden of colon and rectum cancer in 1990 and 2019 for both sexes and all locations, with EAPC.

Characteristics	1990				2019				EAPC (1990–2019)	
	Deaths cases	ASMR per 100,000	DALYs	ASDR per 100,000	Deaths cases	ASMR per 100,000	DALYs	ASDR per 100,000	ASMR	ASDR
	No. (95% UI)	No. (95% UI)	No. (95% UI)	No. (95% UI)	No. (95% UI)	No. (95% UI)	No. (95% UI)	No. (95% UI)	No. (95% CI)	No. (95% CI)
Global	72,199 (44,384–99,966)	1.98 (1.2–2.74)	1,764,172 (1,115,033–2,420,485)	43.71 (27.48–60.09)	166,456 (107,221–226,027)	2.09 (1.34–2.84)	3,799,297 (2,457,768–5,124,453)	46.09 (29.83–62.18)	0.19% (0.16–0.23)	0.19% (0.16–0.22)
*Sex*										
Male	36,470 (22,457–50,700)	2.26 (1.37–3.16)	930,490 (583,525–1,290,972)	49.5 (30.61–68.76)	92,097 (59,298–125,756)	2.56 (1.65–3.51)	2,201,663 (1,426,199–3,006,596)	56.46 (36.57–77.15)	0.48% (0.43–0.54)	0.53% (0.46–0.59)
Female	35,729 (21,869–49,887)	1.76 (1.08–2.47)	833,682 (527,324–1,156,766)	38.9 (24.49–54.09)	74,360 (46,931–99,957)	1.7 (1.07–2.28)	1,597,635 (1,013,555–2,129,091)	36.8 (23.35–49.03)	−0.19% (−0.23 to −0.15)	−1.31% (−1.35 to −1.27)
*SDI region*										
High SDI	26,999 (14,174–39,995)	2.58 (1.36–3.81)	574,929 (306,459–847,756)	56.29 (30.2–82.89)	39,255 (21,762–57,568)	1.95 (1.08–2.85)	734,088 (405,533–1,078,050)	41.4 (22.87–61.09)	−1.14% (−1.2 to −1.09)	−1.25% (−1.3 to −1.19)
High-middle SDI	19,832 (11,648–28,461)	1.98 (1.16–2.84)	484,535 (285,912–693,751)	44.76 (26.41–64.08)	46,115 (28,371–63,889)	2.29 (1.41–3.17)	1,027,477 (642,656–1,415,778)	51 (31.94–70.25)	0.55% (0.49–0.61)	0.5% (0.43–0.56)
Middle SDI	15,910 (11,069–20,548)	1.68 (1.17–2.18)	440,520 (306,405–572,156)	39.54 (27.45–51.13)	52,394 (34,928–69,155)	2.24 (1.49–2.96)	1,300,482 (868,146–1,713,809)	50.78 (34–66.86)	1.13% (1–1.25)	1.02% (0.9–1.14)
Low-middle SDI	6,808 (4,700–9,069)	1.23 (0.85–1.65)	190,759 (130,925–255,206)	29.35 (20.18–39.2)	22,080 (14,880–29,018)	1.72 (1.16–2.25)	561,041 (376,258–741,259)	39.43 (26.46–51.97)	1.13% (1.08–1.18)	1% (0.95–1.04)
Low SDI	2,607 (1,744–3,557)	1.21 (0.81–1.65)	72,422 (48,389–98,926)	28.34 (18.89–38.67)	6,514 (4,308–8,719)	1.39 (0.92–1.86)	173,985 (115,152–233,930)	31.22 (20.64–41.86)	0.46% (0.38–0.54)	0.31% (0.23–0.39)
*GBD regions*										
Andean Latin America	269 (177–367)	1.4 (0.92–1.91)	6,568 (4,323–9,050)	30.67 (20.27–41.98)	998 (629–1,408)	1.83 (1.16–2.58)	21,938 (13,752–31,010)	38.7 (24.22–54.61)	1.13% (0.99–1.28)	1% (0.86–1.15)
Australasia	467 (167–780)	2.02 (0.74–3.38)	10,603 (3,762–17,621)	46.09 (16.5–76.43)	410 (142–806)	0.78 (0.26–1.53)	7,418 (2,234–14,870)	15.82 (4.63–31.74)	−4.1% (−4.54 to −3.65)	−4.58% (−5.09 to −4.08)
Caribbean	580 (376–785)	2.32 (1.51–3.15)	13,271 (8,616–17,842)	50.31 (32.67–67.74)	1,431 (910–1,993)	2.77 (1.76–3.85)	30,710 (19,483–43,044)	59.44 (37.71–83.28)	0.63% (0.58–0.68)	0.61% (0.56–0.66)
Central Asia	481 (232–755)	1.03 (0.5–1.62)	13,946 (6,762–21,821)	27.71 (13.41–43.53)	691 (340–1,098)	1.04 (0.51–1.63)	18,779 (9,253–29,840)	23.81 (11.77–37.7)	−0.09% (−0.28–0.1)	−0.85% (−1.11 to −0.6)
Central Europe	3,711 (1,771–5,620)	2.61 (1.25–3.94)	85,902 (41,312–130,021)	58.38 (28.14–88.38)	6,315 (3,246–9,515)	2.88 (1.49–4.34)	127,691 (66,241–192,342)	62.04 (32.35–93.38)	0.3% (0.18–0.43)	0.15% (0.04–0.26)
Central Latin America	924 (571–1,281)	1.19 (0.73–1.64)	23,316 (14,423–32,353)	26.15 (16.18–36.33)	3,463 (1,972–5,057)	1.5 (0.85–2.19)	82,456 (47,128–121,321)	34.22 (19.49–50.25)	0.78% (0.73–0.82)	0.92% (0.87–0.97)
Central Sub-Saharan Africa	412 (299–546)	2.07 (1.5–2.75)	11,587 (8,322–15,450)	47.27 (34.16–62.7)	929 (632–1,316)	1.98 (1.33–2.84)	25,929 (17,546–36,939)	44.4 (30.15–63.01)	−0.27% (−0.52 to −0.02)	−0.31% (−0.56 to −0.06)
East Asia	15,903 (10,806–20,942)	1.98 (1.35–2.61)	442,322 (299,371–585,149)	46.96 (31.88–61.93)	52,877 (35,241–71,142)	2.7 (1.8–3.61)	1,281,310 (851,746–1,717,804)	61.6 (41.17–82.37)	1.39% (1.15–1.62)	1.28% (1.04–1.51)
Eastern Europe	3,464 (1,195–6,059)	1.28 (0.45–2.21)	86,072 (29,514–150,606)	30.88 (10.73–53.98)	5,912 (2,386–9,539)	1.71 (0.7–2.76)	133,281 (54,087–216,684)	39.85 (16.27–64.62)	0.92% (0.57–1.27)	0.72% (0.32–1.12)
Eastern Sub-Saharan Africa	894 (571–1,253)	1.29 (0.83–1.79)	25,111 (16,002–35,666)	30.55 (19.51–43.06)	2,265 (1,440–3,135)	1.52 (0.97–2.09)	62,468 (39,246–87,119)	34.58 (21.88–47.82)	0.58% (0.51–0.66)	0.43% (0.35–0.51)
High-income Asia Pacific	5,862 (3,680–8,025)	3.06 (1.92–4.17)	137,627 (86,809–188,161)	67.87 (42.77–92.93)	13,124 (8,062–18,133)	2.61 (1.58–3.59)	226,512 (140,680–311,173)	55.14 (34.43–75.73)	−0.65% (−0.71 to −0.59)	−0.85% (−0.92 to −0.78)
High-income North America	7,358 (3,207–11,460)	2.05 (0.9–3.2)	154,202 (66,692–242,683)	45.17 (19.67–70.86)	8,476 (3,297–13,802)	1.32 (0.51–2.16)	175,711 (66,260–288,991)	30.23 (11.48–49.67)	−1.72% (−1.91 to −1.53)	−1.56% (−1.76 to −1.36)
North Africa and Middle East	2,006 (1,240–2,904)	1.27 (0.78–1.84)	54,990 (34,082–79,585)	29.57 (18.3–42.95)	6,185 (3,790–8,725)	1.55 (0.94–2.19)	160,215 (97,499–226,001)	34.47 (20.93–48.64)	0.81% (0.67–0.94)	0.59% (0.46–0.73)
Oceania	39 (25–54)	1.47 (0.95–2.05)	1,134 (735–1,594)	34.17 (22.04–47.92)	104 (66–145)	1.68 (1.09–2.3)	3,017 (1,916–4,272)	38.3 (24.37–53.33)	0.43% (0.36–0.5)	0.37% (0.31–0.43)
South Asia	5,419 (3,766–7,190)	1.07 (0.74–1.42)	151,920 (105,629–202,147)	24.77 (17.15–32.94)	19,002 (12,875–24,981)	1.47 (1–1.93)	474,520 (320,194–626,885)	32.69 (22.04–43.1)	0.94% (0.81–1.06)	0.79% (0.66–0.92)
Southeast Asia	5,071 (3,679–6,466)	2.12 (1.54–2.7)	141,563 (102,790–181,679)	50.47 (36.61–64.3)	16,263 (11,214–21,732)	2.86 (1.97–3.82)	421,003 (287,707–562,038)	65.65 (45.15–87.69)	0.91% (0.85–0.96)	0.78% (0.72–0.84)
Southern Latin America	1,317 (748–1,881)	2.98 (1.68–4.26)	29,110 (16,602–41,794)	62.97 (35.94–90.38)	2,705 (1,542–3,833)	3.2 (1.83–4.54)	55,459 (31,958–78,196)	67.66 (38.95–95.06)	0.26% (0.14–0.37)	0.28% (0.19–0.37)
Southern Sub-Saharan Africa	452 (283–641)	1.8 (1.12–2.57)	11,405 (7,222–15,896)	39.13 (24.76–55.14)	1,017 (637–1,423)	1.98 (1.23–2.76)	25,251 (15,880–35,467)	42.89 (27.02–59.99)	0.32% (0.11–0.53)	0.37% (0.16–0.58)
Tropical Latin America	1,279 (705–1,845)	1.53 (0.84–2.22)	33,040 (18,384–47,517)	34.12 (19–48.94)	3,659 (1,822–5,417)	1.54 (0.77–2.28)	87,061 (43,451–129,380)	35.3 (17.68–52.36)	−0.01% (−0.16–0.14)	0.09% (−0.07–0.26)
Western Europe	15,268 (7,361–23,213)	2.6 (1.25–3.95)	304,737 (147,815–461,611)	54.41 (26.35–82.33)	17,956 (8,091–28,063)	1.8 (0.81–2.84)	311,327 (139,739–493,487)	36.45 (16.41–57.97)	−1.57% (−1.71 to −1.44)	−1.7% (−1.81 to −1.59)
Western Sub-Saharan Africa	1,025 (681–1,431)	1.31 (0.88–1.83)	25,747 (16,853–35,960)	28.4 (18.72–39.7)	2,677 (1,794–3,619)	1.65 (1.1–2.21)	67,242 (44,367–91,855)	34.27 (22.93–46.35)	1.02% (0.91–1.13)	0.86% (0.76–0.96)

ASMR, age-standardized mortality rate; ASDR, age-standardized DALY rate; DALYs, disability-adjusted life-years; EAPC, estimated annual percentage change; SDI, sociodemographic index; UI, uncertainty interval; CI, confidence interval. The above data has been adjusted by DisMod MR version 2.1.

### 3.2 Geographical variations in CRC attributable to diet low in milk

In 2019, the middle SDI regions had the highest number of CRC-related deaths [52,394 (95% UI = 34,928–69,155)] and DALYs [1,300,482 (95% UI = 868,146–1,713,809)] due to diet low in milk. Conversely, the lowest SDI regions had the low number of CRC-related deaths [6,514 (95% UI = 4,308–8,719)] and DALYs [173,985 (95% UI = 115,152–233,930)]. At the same time, high-middle SDI regions had the greatest ASMR [2.29 (95% CI = 1.41–3.17)] and ASDR [51 (95% CI = 31.94–70.25)] per 100,000, respectively. From 1990 to 2019, the ASMR and ASDR of the low, low-middle, middle, and high-middle SDI regions had gradually increased, whereas those of the high SDI regions had decreased (Table 1).

Geographically, East Asia had the highest number of CRC-related deaths [52,877 (95% UI = 35,241–71,142)] and DALYs [1,281,310 (95% UI = 851,746–1,717,804)] in 2019 associated with diet low in milk. Also in 2019, Australasia had the lowest and Southern Latin America had the highest ASMR and ASDR (0.78 and 3.2 deaths vs. 15.82 and 67.66 DALYs per 100,000, respectively). Between 1990 and 2019, Australasia had the largest decreases and East Asia had the largest increases in ASMR and ASDR [−4.1 (95% CI = −4.54 to −3.65) vs. 1.39 (95% CI = 1.15–1.62) and −4.58 (95% CI = −5.09 to −4.08) vs. 1.28 (95% CI = 1.04–1.51), respectively] (Table 1). Central Sub-Saharan Africa, Southeast Asia, and South Asia had the largest percentage of CRC deaths and DALYs linked to low milk consumption in 1990, while Eastern Europe, Australasia, Central Asia and High-income North America had the lowest percentages. This disparity was roughly three times larger in 1990. Similar trends were observed in the proportion attributable to diet low in milk in 2019 (Figure 1).

### 3.3 Country-level burden of CRC attributable to diet low in milk

**Figure 3:**
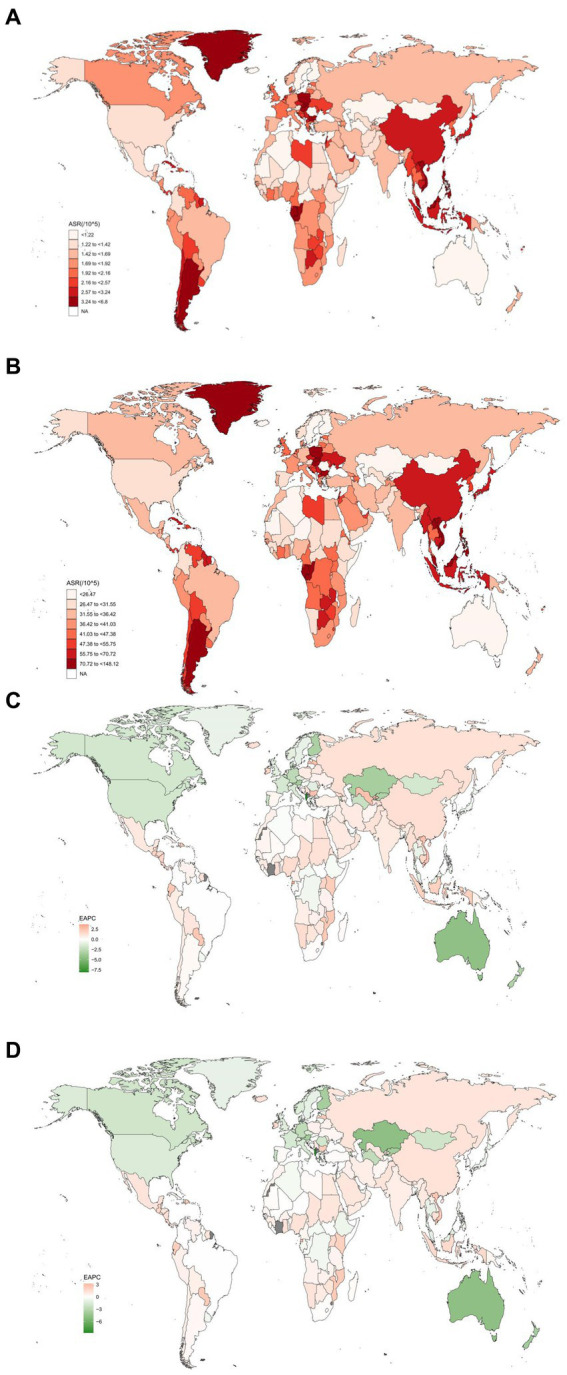
The spatial distribution of the colorectal cancer ASMR **(A)** and ASDR **(B)** attributable to diet low in milk in 2019, and the EAPC in colorectal cancer ASMR **(C)** and ASDR **(D)** attributable to diet low in milk. ASMR, age-standardized mortality rate; ASDR, age-standardized DALY rate; EAPC, estimated annual percentage change.

In 2019, at the nation level, China had the highest number of CRC deaths and DALYs associated with diet low in milk [50,310 (95% UI = 33,195–67,665) and 1,222,433 (95% UI = 804,709–1,648,733), respectively], followed by India [16,394 (95% UI = 11,214–21,686) and 406,643 (95% UI = 276,849–542,329), respectively], and Japan [10,794 (95% UI = 6,541–1,5,041) and 178,533 (95% UI = 109,415–248,995), respectively]. Also in 2019, Albania, Finland, and Kyrgyzstan had the greatest ASMR owing to diet low in milk, whereas Seychelles, Brunei Darussalam, and Taiwan (Province of China) had the highest ASDR (Figures 3A,B). Equatorial Guinea, Latvia, and Vietnam had the highest increases in ASMR, with EAPCs of 3.67 (95% CI = 3.37–3.96), 2.68 (95% CI = 1.89–3.48), and 2.65 (95% CI = 2.42–2.67), respectively. Similarly, Equatorial Guinea, Lesotho, and Vietnam had the highest increases in ASDR, with EAPCs of 3.22 (95% CI = 2.95–3.49), 2.64% (95% CI = 2.41–2.87), and 2.57 (95% CI = 2.42–2.72), respectively. Meanwhile, Albania experienced the fastest decline in ASMR and ASDR, with EAPCs of −8.06 (95% CI = −8.74 to −7.38) and −8.74 (95% CI = −9.56 to −7.91), respectively (Figures 3C,D; Supplementary Tables S1–S6). The percentage change in the fraction of all CRC deaths and DALYs attributable to diet low in milk is shown in Supplementary Tables S7, S8.

### 3.4 Global CRC burden attributable to diet low in milk by sex and age

Globally, sex inequality continues to influence the burden of CRC attributable to diet low in milk, with a greater impact on males, which increased with age. In 2019, CRC-related deaths and DALYs were more common in males than females [92,097 (95% UI = 59,298–125,756) vs. 74,360 (95% UI = 46,931–99,957) and 2,201,663 (95% UI = 1,426,199–3,006,596) vs. 1,597,635 (95% UI = 1,013,555–2,129,091), respectively] (Table 1). In 2019, the ASMR per 100,000 of CRC linked to diet low in milk was greater in males than females [2.56 (95% UI = 1.65–3.51) vs. 1.7 (95% UI = 1.07–2.28), respectively]. Similarly, ASDR was greater in males than females [56.46 (95% UI = 36.57–77.15) vs. 36.8 (95% UI = 23.35–49.03), respectively]. From 1990 to 2019, the average annual increase in ASMR and ASDR of CRC attributable to diet low in milk was 0.48 (95% CI = 0.43–0.54) and 0.53 (95% CI = 0.46–0.59) for males, while the average annual decrease was −0.19 (95% CI = −0.23 to −0.15) and −1.31 (95% CI = −1.35 to −1.27) for females.

**Figure 4:**
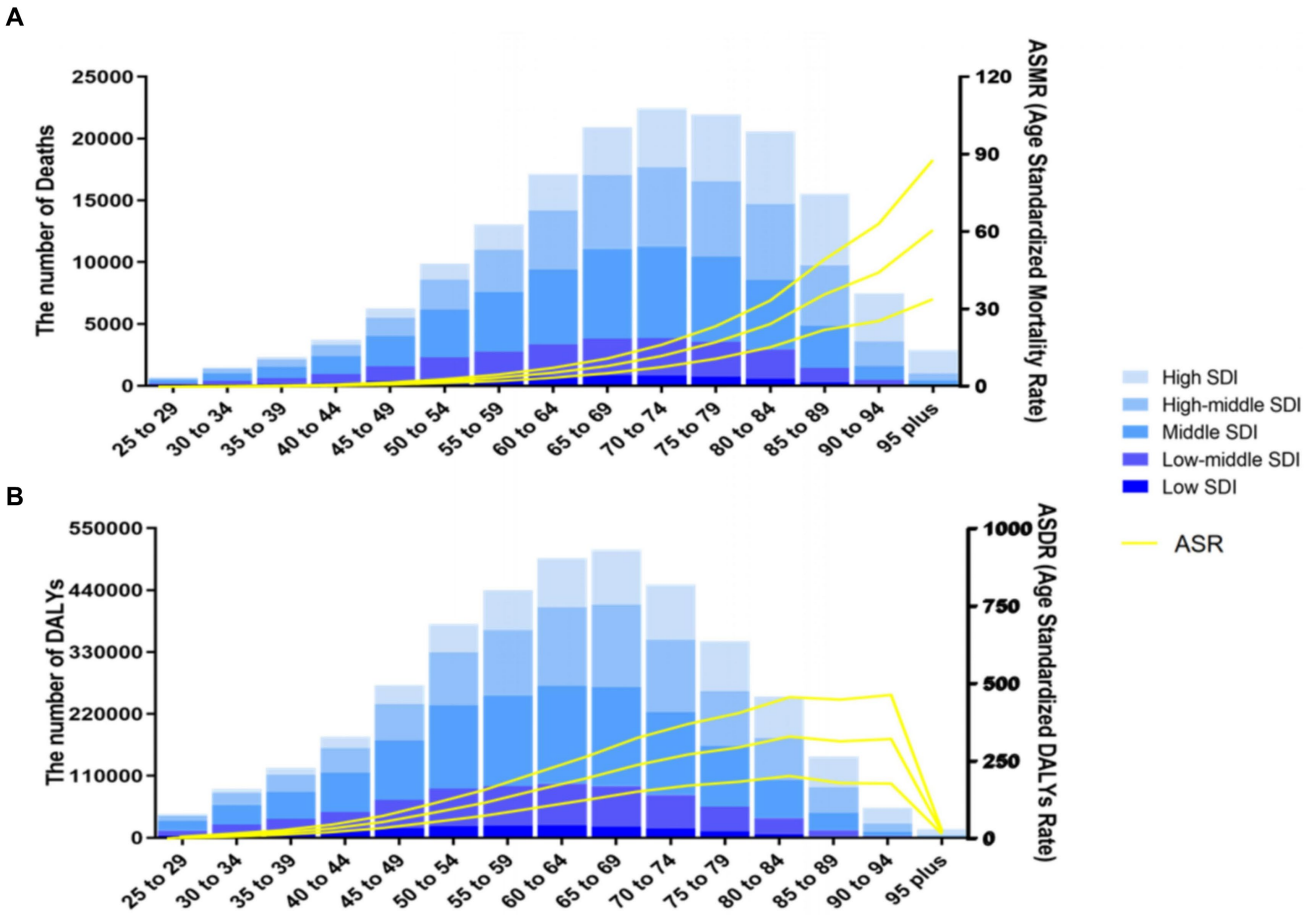
Number and rate of colorectal cancer deaths **(A)** and DALYs **(B)** attributable to diet low in milk by age group and SDI level in 2019. The bars represent the number of colorectal cancer deaths **(A)** and DALYs **(B)** attributable to diet low in milk colored by SDI level. The line represents the mean ASMR **(A)** and ASDR **(B)** (per 100,000) attributable to diet low in milk at the global level. The shaded area represents the 95% UI for the mean rate. DALYs, disability-adjusted life-years; ASMR, age-standardized mortality rate; ASDR, age-standardized DALY rate; UI, uncertainty interval; SDI, socio-demographic index.

**Figure 5:**
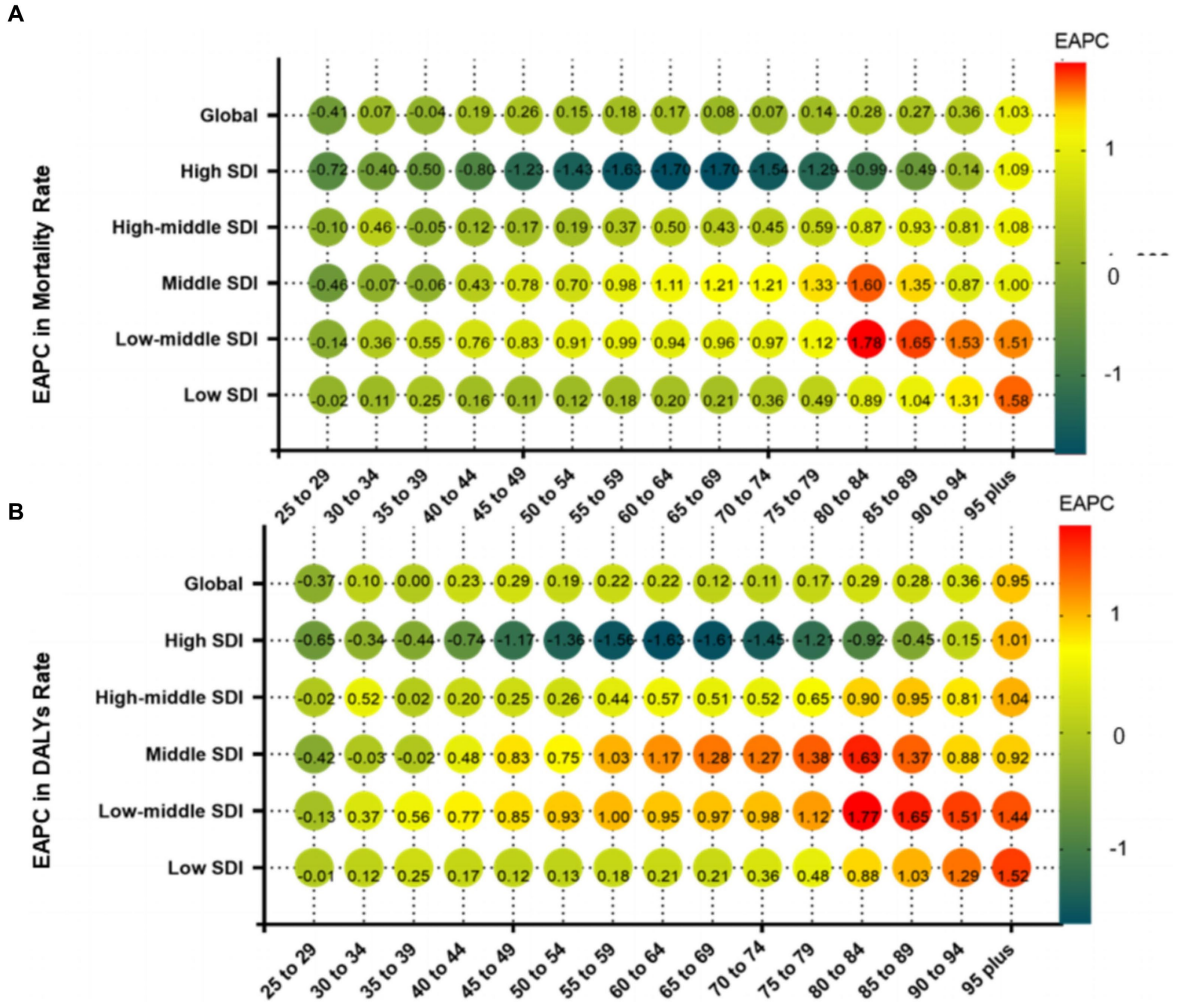
Annual percentage change in mortality **(A)** and DALYs **(B)** between 1990 and 2019 by age group and region. EAPC, estimated annual percentage change; SDI, socio-demographic index; DALYs, disability-adjusted life-years.

In 2019, the ASMR of CRC attributable to diet low in milk increased with age, with the largest number of deaths occurring in those aged 70–74 years (Figure 4A). Meanwhile, the ASDR decreased progressively after peaking in the group aged 90–94 years, and the largest number of DALYs in the group aged 65–69 years (Figure 4B). From 1990 to 2019, ASMR increased globally in almost all age groups, except for a decrease in groups aged 25–29 and 35–39 years. The group aged >95 years experienced the greatest growth, while the group aged 25–29 had the greatest decline. The ASMR increased in low, low-middle, middle and high-middle SDI regions from 1990 to 2019, with higher EAPCs in mortality in low-middle SDI regions (Figure 5A). The age-standardized DALY rates were similar for EAPCs and ASMR (Figure 5B). The distribution of deaths and DALYs remained stable over time in each of the four age subgroups (Supplementary Figure S1).

Cluster analysis revealed that 121 countries or territories, most notably North Macedonia, Benin, and Burkina Faso, were classified as having “remained stable,” while 20 countries or territories were classified as “minor increase,” which included Singapore, Israel, and Portugal. Additionally, 56 countries or territories, including Paraguay, the Dominican Republic, and Mozambique, were classified as “increase.” Albania was the only country to be classified as “significant decrease.” The remaining six nations or territories, including Finland, Kazakhstan, and Australia, were classified as “decrease” (Supplementary Figure S2).

### 3.5 Factors associated with the burden of CRC burden attributable to diet low in milk

**Figure 6:**
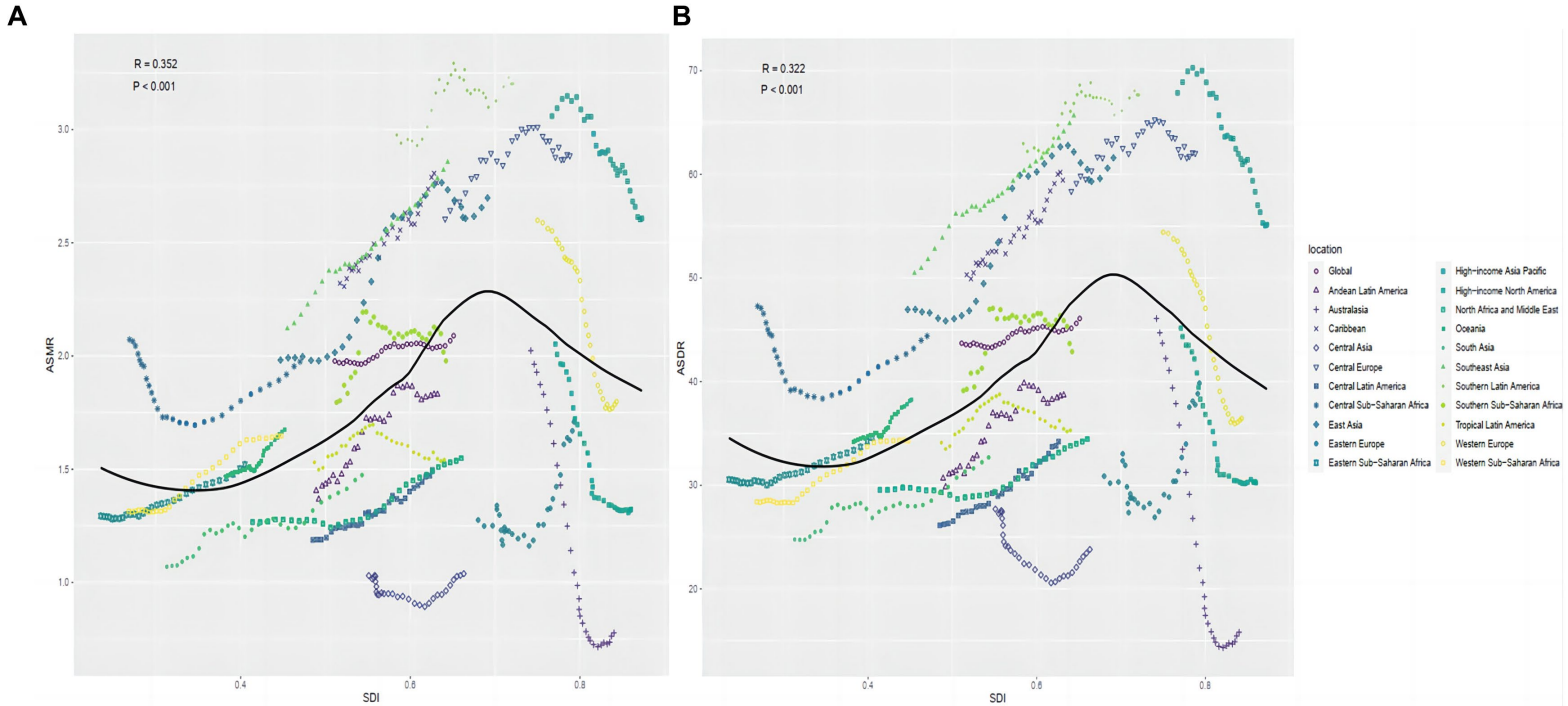
Correlation between diet low in milk-attributable colorectal cancer in ASMR **(A)** or ASDR **(B)** and SDI globally in 21 GBD regions between 1990 and 2019. ASMR, age-standardized mortality rate; ASDR, age-standardized DALY rate; GBD, global burden of disease study.

**Figure 7:**
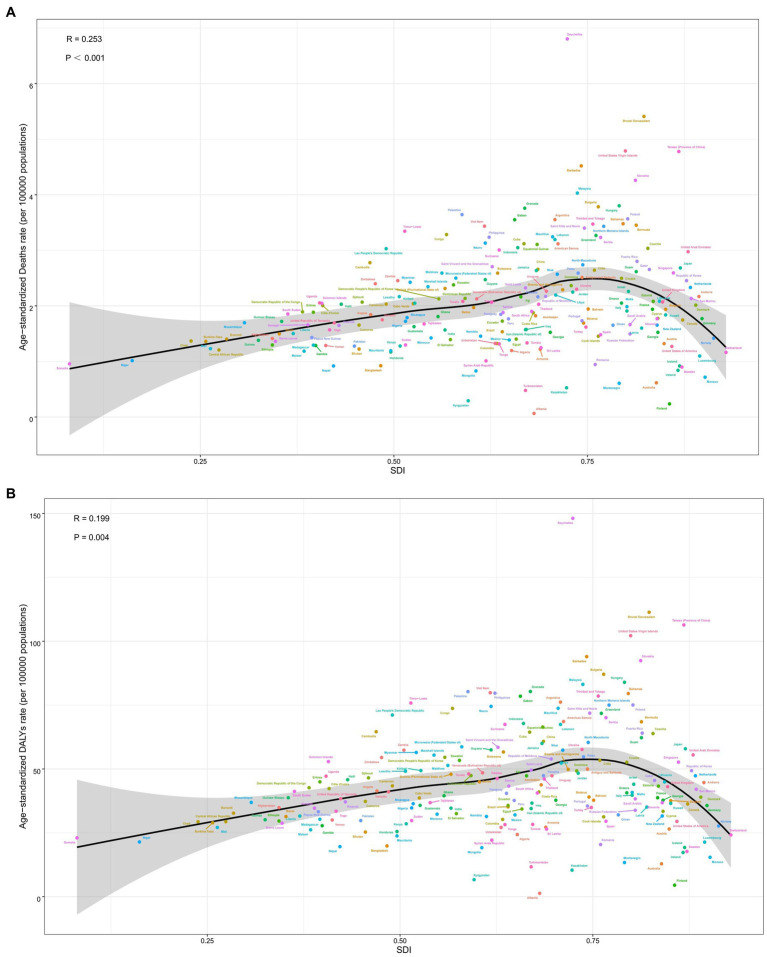
Correlation between diet low in milk attributable to colorectal cancer in ASMR **(A)**, ASDR **(B)** and SDI globally in 204 nations. ASMR, age-standardized mortality rate; ASDR, age-standardized DALY rate; SDI, socio-demographic index.

Overall, there was a nonlinear “S”-shaped association between the overall ASMR and SDI, with the ASMR rapidly increasing when the SDI was greater than 0.75 and progressively decreasing at less than 0.45. Among the different regions, the highest ASMR related to diet low in milk were observed in Southern Latin America, followed by high-income Asia Pacific and Central Europe, while the lowest ASMR and ASDR related to diet low in milk occurred in Australasia and Central Asia (Figure 6A). A similar association occurred between ASDR and SDI (Figure 6B). In 2019, across 204 countries and territories globally, the relationships between ASMR and ASDR attributable to CRC and SDI initially increased and then decreased as SDI increased (Figures 7A,B).

**Figure 8:**
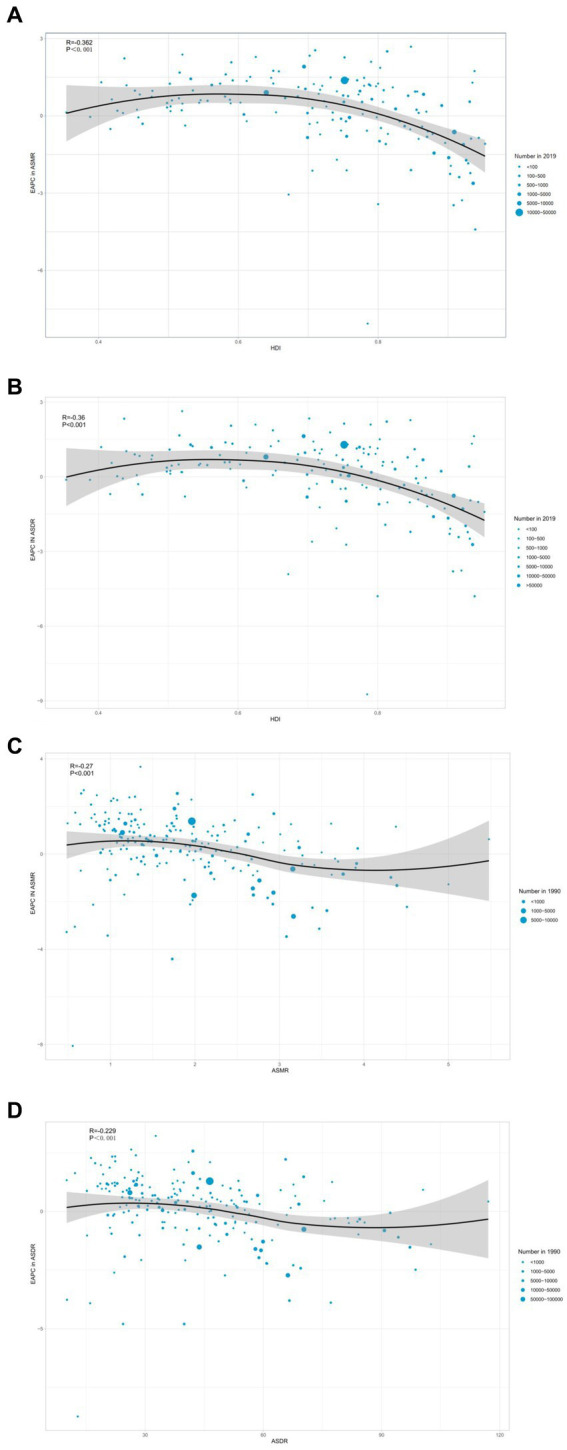
Correlation between EAPC in ASMR **(A)**, ASDR **(B)** and HDI in 2019. Correlation between EAPC in ASMR and ASMR in 1990 **(C)**. Correlation between EAPC in ASDR and ASDR in 1990 **(D)**. EAPC, estimated annual percentage change; ASMR, age-standardized mortality rate; HDI, human development index; ASDR, age-standardized DALY rate.

A noteworthy negative correlation (*R* = −0.362, *p* < 0.001) was observed in 2019 between the EAPC in ASMR and HDI (Figure 8A). Similarly, an inverse relationship (*R* = −0.36, *p* < 0.001) occurred between the EAPC in ASDR and HDI in 2019 across various countries (Figure 8B). The same pattern occurred with the EAPC in ASMR and ASMR in 1990 (*R* = −0.27, *p* < 0.001) (Figure 8C), as well as the EAPC in ASDR and ASDR in 1990 (*R* = −0.229, *p* < 0.001) (Figure 8D).

## 4 Discussion

This analysis comprehensively summarized the global epidemiological trends of CRC attributable to diet low in milk. The findings showed that incidences of deaths and DALYs attributable to CRC were 15.3 and 18.6%, respectively. On a global scale, the number of CRC-related deaths and DALYs attributable to diet low in milk increased by 130.5 and 115.4% from 1990 to 2019, respectively. The spatial distribution of the burden of CRC varied significantly among different countries and regions. The ASMR and ASDR associated with CRC attributable to diet low in milk were more significant in high-middle SDI regions. East Asia, especially China, had the highest number of CRC-related deaths and DALYs attributable to diet low in milk. The burden of CRC was higher in males than females and the elderly than younger populations. These findings filled the gap in the global burden of CRC attributable to diet low in milk, help raise awareness of the importance of increasing milk intake, and provided evidence for policy makers to adapt appropriate dietary strategies to better manage CRC patients.

Diet low in milk is the leading risk factor for the burden of CRC worldwide. A prior study indicated that individuals who consumed ≥250 g of milk per day had a 15% lower risk of CRC as compared to those who consumed <70 g, and each increase of 500 g per day in milk intake reduced the risk of CRC by 12% (19). However, the mechanism underlying the reduced risk of CRC associated with increased milk consumption remains unclear. As a possible explanation, milk contains a large amount of calcium, which may be responsible for the reported inverse association between milk intake and CRC. Through colonic sequestration of secondary bile acids, including deoxycholic acid and phospholipids, calcium could provide protection against CRC (20, 21). Moreover, additional nutrients or bioactive substances found in milk, such as vitamin D, lactoferrin, and the short-chain fatty acid butyrate, might also act to prevent CRC (22, 23). Alarmingly, the number of individuals who consume an insufficient amount of milk has increased significantly (24). Globally, there was a substantial disparity between the current and optimal milk intake in 2017, with an average optimal consumption of 16% (25). The 2020–2025 Dietary Guidelines for Americans recommend 3 cup equivalent servings of skim or semiskim milk daily for all adults. However, adults aged ≥20 years consume only 1.5 cup-equivalents of dairy products daily (26). The major reasons for this are rapid economic development and changing dietary patterns, as milk consumption has decreased in favor of sweetened beverages and fruit juices. Sweetened beverages include regular sweetened carbonated soda, sports drinks, energy drinks, and non-pure fruit drinks. Sugar beverages and fruit juices often contain high levels of added sugars, which could potentially contribute to weight gain, inflammation, and metabolic dysregulation, all of which are implicated in CRC development (27). Due to the complexity of diet, these substitutes may confound or interact with the relationship between milk consumption and colorectal cancer risk. It is necessary to conduct further in-depth research on the complex interactions of dietary risks in the future. In addition, education and food security have also been associated with insufficient milk intake. So, education and awareness campaigns should be launched to encourage daily consumption of milk and other dairy products. Variable correlations may exist between the risk of CRC and the consumption of whole, skim, and semiskim milk and related fat components (28). The consumption of whole milk was positively linked with CRC mortality, while consumption of skim milk was negatively associated with CRC mortality, possibly due to fat-induced inhibition of other bioactive components in skim milk (29, 30). However, the GBD database did not further classify milk types, so the impact of different types of milk deficiency on the burden of CRC remains unclear.

This study investigated sex differences in the incidence of CRC attributable to diet low in milk. Since 1990, the increases in ASDR and ASMR were more pronounced in males than females with a notable difference in the contribution of diet low in milk to CRC (11.1% vs. 4.6%, respectively) (31). As a potential explanation for this finding, women are more likely to receive recommendations to increase milk intake than men because of the greater risk of osteoporosis (32, 33). Furthermore, women tend to be more concerned and consciousness of health status. Sex hormones have been recognized as a factor in sex differences in the incidence and mortality of CRC (34). Furthermore, risk behaviors, including drinking alcohol and smoking, are more common in men (35). Milk consumption decreases in the elderly due to various factors, such as decreased concern for personal health, higher rates of milk intolerance and digestion problems, changes in dietary preferences attributable to taste and palatability, and efforts to reduce fat intake (36). Therefore, initiatives are needed to increase awareness of milk intake to reduce sex-based disparities and decrease the incidence of preventable cancers.

There was an “S”-shaped correlation between ASMR or ASDR attributable to diet low in milk and SDI worldwide. The study revealed that diet low in milk remained common in Central sub-Saharan Africa, Southeast Asia, and South Asia. Conversely, milk intake has dramatically increased in North America, Central Asia, and Australasia, especially Australia (7). The primary factors contributing to these geographical disparities include behaviors related to lifestyle and diet, levels of socioeconomic development, and local medical conditions (37, 38). With the regional development, increased household income, and improvements in education, the consumption frequency and daily intake of milk have increased (39). For example, daily milk intake ranged from <200 g to >600 g in Western populations and from <42.4 g to >82.6 g in Asian populations (40). Notably, lactose intolerance is the major cause of milk restriction globally (41). The prevalence of lactose intolerance exhibits racial variation, from 64% in Asia to as low as 28% in Northern Europe (42). Even though it is difficult to encourage immediate change to dietary habits, education and propaganda campaigns are recommended to gradually increase daily milk intake.

China is the most populous nation globally and also has the highest number of CRC-related deaths and DALYs attributable to diet low in milk (43). Consumption of milk and dairy products continues to increase in China, but remains relatively low due to traditional dietary habits (44). A comprehensive prospective study reported that the average daily intake of milk and dairy products in China has increased from 2.06 g in 1989 to 26.47 g in 2011, which is still significantly lower than in European and North American countries (39). The recommended intake of milk and dairy products was revised in the latest version of the Chinese Dietary Guidelines for Residents (2022), from no less than 300 g per day to 300–500 g per day. Colonoscopy is considered the gold standard for CRC screening. However, due to a lack of a comprehensive national screening program and health resource restrictions, population-based screening of CRC has not yet been implemented in China (45).

As this study is a longitudinal observational study, although the modeling approach used estimates risk based on available dietary risk data but does not establish causation and should be regarded as approximations of risk (46). The GBD study had some deficiencies. Primarily, underdeveloped countries lack adequate cancer registries, thus estimates were based on predictive covariates or trends in neighboring countries, which likely biased the results. Second, the impact of different types of milk deficiencies on the burden of CRC remain unclear due to the lack of relevant data. Furthermore, the interrelationship between diets may have influenced the estimated burden of CRC attributable to diet low in milk. Although many of these dietary relative risks have been adjusted for the major confounders (e.g., age, sex), the possibility of residual confounding cannot be excluded.

## 5 Conclusion

This study comprehensively analyzed the association between the global burden of CRC and diet low in milk. The results indicated that the number of CRC-related deaths and DALYs due to diet low in milk continued to increase globally. There were significant differences in the burden of CRC linked to diet low in milk among countries and regions. Notably, high-middle SDI regions had the highest ASDR and ASMR of CRC attributable to diet low in milk. East Asia, especially China, had the highest number of CRC-related deaths and DALYs attributable to diet low in milk. In addition, diet low in milk has been associated with a greater burden of CRC in males and older individuals. These findings provided critical temporal and geographic data to assist policymakers to develop targeted dietary strategies for CRC patients, as well as raise public awareness regarding the necessity of increasing milk intake. Due to the interrelationships between diets and potential confounding factors that may affect the estimated burden of CRC attributed to diet low in milk, further research is needed in the future to understand the complexity of dietary alternatives.

## Data availability statement

The datasets presented in this study can be found in online repositories. The names of the repository/repositories and accession number(s) can be found in the article/Supplementary material.

## Author contributions

XgZ: Writing – original draft, Writing – review & editing, Data curation, Methodology, Project administration, Supervision. XrZ: Data curation, Writing – original draft, Writing – review & editing. RL: Data curation, Writing – original draft, Writing – review & editing. ML: Data curation, Writing – original draft, Writing – review & editing. TO: Data curation, Writing – original draft, Writing – review & editing. HZ: Data curation, Writing – original draft, Writing – review & editing. ZC: Project administration, Writing – original draft, Writing – review & editing, Supervision. LZ: Project administration, Writing – original draft, Writing – review & editing, Supervision.

## Footnotes

^1^ http://ghdx.healthdata.org/gbd-results-tool

^2^ http://hdr.undp.org/en/data

^3^ https://www.r-project.org/

## Supplementary material

SUPPLEMENTARY FIGURE S1 Age composition ratio of colorectal cancer deaths and DALYs attributable to diet low in milk, 1990–2019.

SUPPLEMENTARY FIGURE S2 Hierarchical cluster analysis based on national and regional temporal trends in the ASMR and ASDR.

SUPPLEMENTARY TABLE S1 Top 10 countries or territories with the highest number of colorectal cancer deaths attributable to diet low in milk in 2019.

SUPPLEMENTARY TABLE S2 Top 10 countries or territories with the highest number of colorectal cancer DALYs related to diet low in milk in 2019.

SUPPLEMENTARY TABLE S3 Top 10 countries or territories with the highest colorectal cancer ASMR (per 100,000) attributable to diet low in milk in 2019.

SUPPLEMENTARY TABLE S4 Top 10 countries or territories with the highest colorectal cancer ASDR (per 100,000) attributable to diet low in milk in 2019.

SUPPLEMENTARY TABLE S5 Top 10 countries or territories with the highest or lowest EAPC in the ASMR (per 100,000) attributable to diet low in milk, 1990–2019.

SUPPLEMENTARY TABLE S6 Top 10 countries or territories with the highest or lowest EAPC in the ASDR (per 100,000) attributable to diet low in milk, 1990–2019.

SUPPLEMENTARY TABLE S7 Percentage change across countries of the fraction of all colorectal cancer deaths attributable to diet low in milk (95% CI).

SUPPLEMENTARY TABLE S8 Percentage change across countries of the fraction of all colorectal cancer DALYs attributable to diet low in milk (95% CI).
