# Supplementary figures and images for: Global, regional, and national analyses of the burden of colorectal cancer attributable to diet low in milk from 1990 to 2019: longitudinal observational study
Source: Front Nutr. 2024 Jul 22;11:1431962. doi: 10.3389/fnut.2024.1431962 (PMC11299434; doi:10.3389/fnut.2024.1431962)

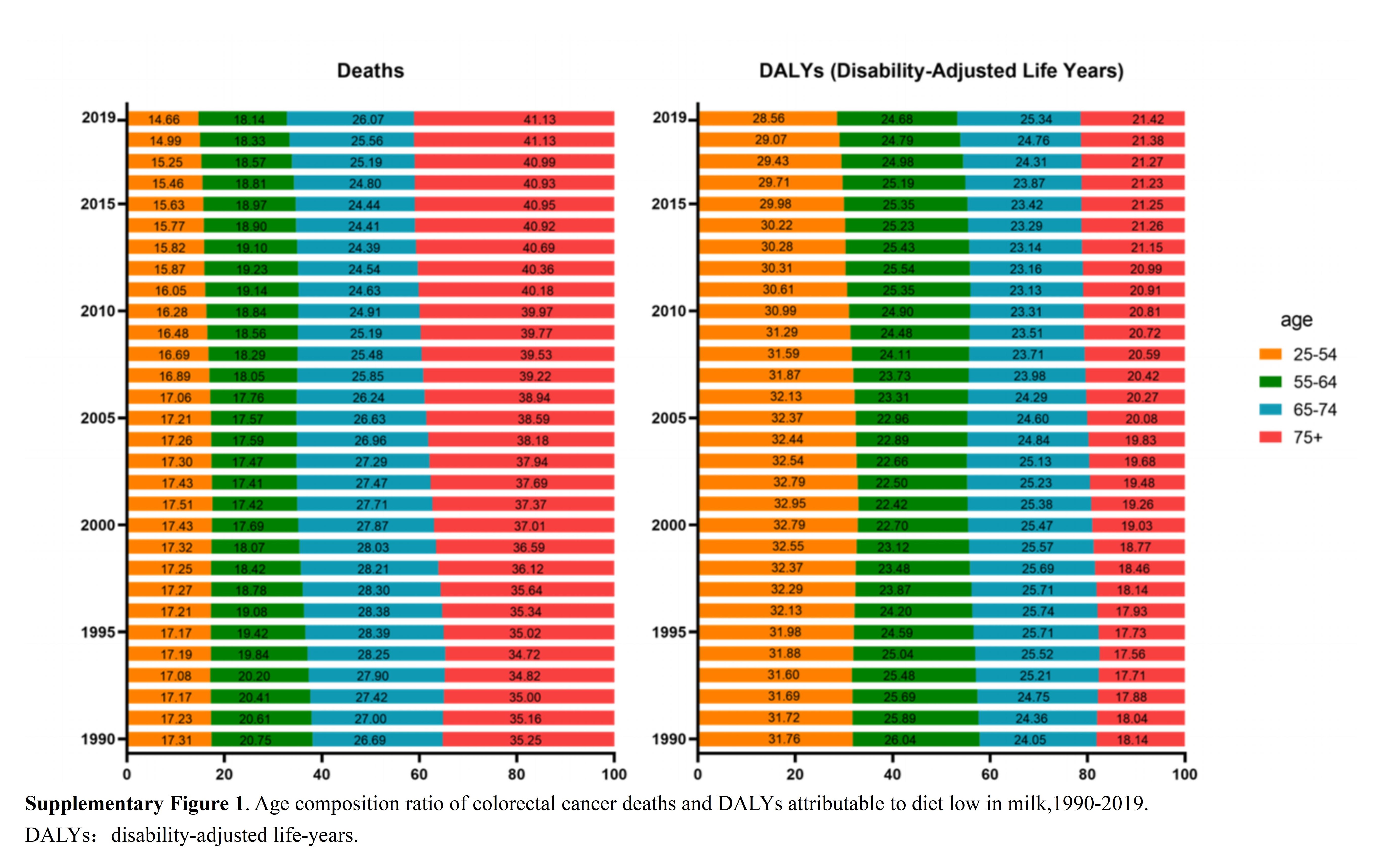

Supplement: SUPPLEMENTARY FIGURE S1 — Age composition ratio of colorectal cancer deaths and DALYs attributable to diet low in milk, 1990–2019. [file Image_1.JPEG]

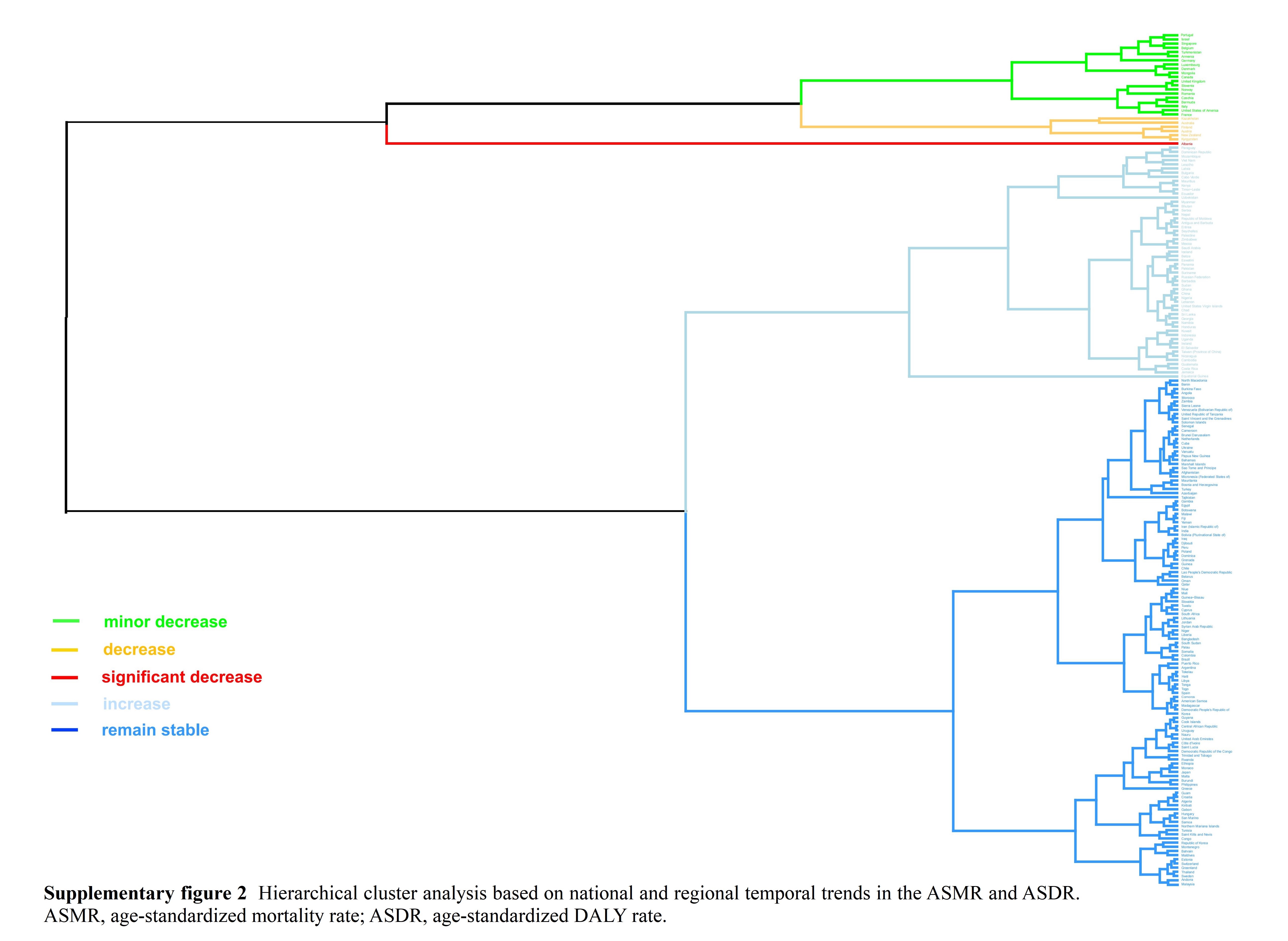

Supplement: SUPPLEMENTARY FIGURE S2 — Hierarchical cluster analysis based on national and regional temporal trends in the ASMR and ASDR. [file Image_2.JPEG]
