# Supplementary material for: Global, regional, and national analyses of the burden of colorectal cancer attributable to diet low in milk from 1990 to 2019: longitudinal observational study
Source: Front Nutr. 2024 Jul 22;11:1431962. doi: 10.3389/fnut.2024.1431962 (PMC11299434; doi:10.3389/fnut.2024.1431962)
Supplement: SUPPLEMENTARY TABLE S1 — Top 10 countries or territories with the highest number of colorectal cancer deaths attributable to diet low in milk in 2019. [file Table_1.docx]

| **Supplementary Table 1.** Top 10 countries or territories with the highest number of colorectal cancer Deaths related to diet low in milk in 2019. | |
| --- | --- |
| **Location** | **No. (95% UI)** |
| China | 50310(33195 , 67665) |
| India | 16394(11214 , 21686) |
| Japan | 10794(6541 , 15041) |
| United States of America | 7211(2752 , 11837) |
| Indonesia | 5903(3543 , 8518) |
| Germany | 3679(1405 , 5868) |
| Brazil | 3560(1768 , 5262) |
| Russian Federation | 3517(1257 , 5828) |
| Viet Nam | 3053(2024 , 4311) |
| France | 3028(1370 , 4695) |

UI: uncertainty interval. The above data has been adjusted by DisMod MR version 2.1.
