# Supplementary material for: Global, regional, and national analyses of the burden of colorectal cancer attributable to diet low in milk from 1990 to 2019: longitudinal observational study
Source: Front Nutr. 2024 Jul 22;11:1431962. doi: 10.3389/fnut.2024.1431962 (PMC11299434; doi:10.3389/fnut.2024.1431962)
Supplement: SUPPLEMENTARY TABLE S2 — Top 10 countries or territories with the highest number of colorectal cancer DALYs related to diet low in milk in 2019. [file Table_2.docx]

| **Supplementary Table 2** Top 10 countries or territories with the highest number of colorectal cancer DALYs related to diet low in milk in 2019. | |
| --- | --- |
| **Location** | **No. (95% UI)** |
| China | 1222433(804709 , 1648733) |
| India | 406643(276849 , 542329) |
| Japan | 178533(109415 , 248995) |
| Indonesia | 155821(94364 , 224988) |
| United States of America | 151912(57205 , 250604) |
| Brazil | 84752(42475 , 125796) |
| Viet Nam | 78807(51867 , 112747) |
| Russian Federation | 77572(27349 , 129913) |
| Philippines | 68430(46788 , 92598) |
| Germany | 63685(23958 , 102062) |

DALYs: disability-adjusted life-years. UI: uncertainty interval.The above data has been adjusted by DisMod MR version 2.1.
