# Supplementary material for: Global, regional, and national analyses of the burden of colorectal cancer attributable to diet low in milk from 1990 to 2019: longitudinal observational study
Source: Front Nutr. 2024 Jul 22;11:1431962. doi: 10.3389/fnut.2024.1431962 (PMC11299434; doi:10.3389/fnut.2024.1431962)
Supplement: SUPPLEMENTARY TABLE S3 — Top 10 countries or territories with the highest colorectal cancer ASMR (per 100,000) attributable to diet low in milk in 2019. [file Table_3.docx]

| **Supplementary Table 3.** Top 10 countries or territories with the highest colorectal cancer ASMR (per 100 000) attributable to diet low in milk in 2019. | |
| --- | --- |
| **Location** | **No. (95% UI)** |
| Ireland | 0.84(0.22,1.71) |
| Mongolia | 0.83(0.28,1.41) |
| Monaco | 0.72(0.16,1.81) |
| Australia | 0.62(0.17,1.36) |
| Montenegro | 0.61(0.16,1.38) |
| Kazakhstan | 0.53(0.18,1.11) |
| Turkmenistan | 0.48(0.15,0.88) |
| Kyrgyzstan | 0.29(0.07,0.66) |
| Finland | 0.24(0.05,0.67) |
| Albania | 0.06(0.02,0.21) |

ASMR: age-standardized mortality rate.UI: uncertainty interval.The above data has been adjusted by DisMod MR version 2.1.
