# Supplementary material for: Global, regional, and national analyses of the burden of colorectal cancer attributable to diet low in milk from 1990 to 2019: longitudinal observational study
Source: Front Nutr. 2024 Jul 22;11:1431962. doi: 10.3389/fnut.2024.1431962 (PMC11299434; doi:10.3389/fnut.2024.1431962)
Supplement: SUPPLEMENTARY TABLE S4 — Top 10 countries or territories with the highest colorectal cancer ASDR (per 100,000) attributable to diet low in milk in 2019. [file Table_4.docx]

| **Supplementary Table 4.** Top 10 countries or territories with the highest colorectal cancer ASDR (per 100 000) attributable to diet low in milk in 2019. | |
| --- | --- |
| **Location** | **No. (95% UI)** |
| Seychelles | 148.12(113.44,184.95) |
| Brunei Darussalam | 111.35(70.89,154.04) |
| Taiwan (Province of China) | 106.37(65.02,157.58) |
| United States Virgin Islands | 102.18(64.62,147.4) |
| Barbados | 93.97(60.9,130.14) |
| Slovakia | 92.4(52.28,139.95) |
| Bulgaria | 87.07(45.66,136.93) |
| Hungary | 83.93(40.03,132.7) |
| Malaysia | 83.11(54.76,115.45) |
| Grenada | 80.33(55.16,105.89) |

ASDR: age-standardized DALY rate. UI: uncertainty interval.The above data has been adjusted by DisMod MR version 2.1.
