# Supplementary material for: Global, regional, and national analyses of the burden of colorectal cancer attributable to diet low in milk from 1990 to 2019: longitudinal observational study
Source: Front Nutr. 2024 Jul 22;11:1431962. doi: 10.3389/fnut.2024.1431962 (PMC11299434; doi:10.3389/fnut.2024.1431962)
Supplement: SUPPLEMENTARY TABLE S5 — Top 10 countries or territories with the highest or lowest EAPC in the ASMR (per 100,000) attributable to diet low in milk, 1990–2019. [file Table_5.docx]

| **Supplementary Table 5.** Top 10 countries or territories with the highest or lowest EAPC in the ASMR (per 100 000) attributable to diet low in milk，1990–2019. | |
| --- | --- |
| **Location** | **No. (95% CI)** |
| Equa,rial Guinea | 3.67%(3.37,3.96) |
| Latvia | 2.68%(1.89,3.48) |
| Viet Nam | 2.55%(2.42,2.67) |
| Uzbekistan | 2.54%(2.39,2.7) |
| Bulgaria | 2.5%(2,3) |
| Cabo Verde | 2.47%(2.13,2.81) |
| Dominican Republic | 2.41%(2.21,2.61) |
| Lesotho | 2.38%(2.2,2.56) |
| Paraguay | 2.33%(2.14,2.53) |
| Timor-Leste | 2.29%(2.05,2.53) |
| Albania | -8.06%(-8.74,-7.38) |
| Australia | -4.41%(-4.78,-4.04) |
| Austria | -3.47%(-3.69,-3.25) |
| Kazakhstan | -3.43%(-4.11,-2.74) |
| Finland | -3.28%(-3.72,-2.84) |
| New Zealand | -3.14%(-3.89,-2.38) |
| Kyrgyzstan | -3.06%(-3.35,-2.76) |
| Germany | -2.62%(-3.18,-2.05) |
| Belgium | -2.38%(-2.64,-2.11) |
| Israel | -2.25%(-2.54,-1.96) |

ASMR: age-standardized mortality rate. EAPC: estimated annual percentage change.CI: confidence interval.The above data has been adjusted by DisMod MR version 2.1.
