# Supplementary material for: Global, regional, and national analyses of the burden of colorectal cancer attributable to diet low in milk from 1990 to 2019: longitudinal observational study
Source: Front Nutr. 2024 Jul 22;11:1431962. doi: 10.3389/fnut.2024.1431962 (PMC11299434; doi:10.3389/fnut.2024.1431962)
Supplement: SUPPLEMENTARY TABLE S6 — Top 10 countries or territories with the highest or lowest EAPC in the ASDR (per 100,000) attributable to diet low in milk, 1990–2019. [file Table_6.docx]

| **Supplementary Table 6.** Top 10 countries or territories with the highest or lowest EAPC in the ASDR (per 100 000) attributable to diet low in milk, 1990–2019. | |
| --- | --- |
| **Location** | **No. (95% CI)** |
| Equa,rial Guinea | 3.22%(2.95,3.49) |
| Lesotho | 2.64%(2.41,2.87) |
| Viet Nam | 2.57%(2.42,2.72) |
| Dominican Republic | 2.39%(2.23,2.55) |
| Paraguay | 2.34%(2.16,2.53) |
| Mozambique | 2.33%(2.16,2.5) |
| Latvia | 2.27%(1.49,3.07) |
| Bulgaria | 2.22%(1.74,2.7) |
| Ecuador | 2.14%(1.84,2.44) |
| Timor-Leste | 2.1%(1.85,2.34) |
| Albania | -8.74%(-9.56,-7.91) |
| Kazakhstan | -4.8%(-5.67,-3.92) |
| Australia | -4.8%(-5.2,-4.4) |
| Kyrgyzstan | -3.91%(-4.27,-3.55) |
| New Zealand | -3.89%(-4.81,-2.96) |
| Austria | -3.8%(-4.06,-3.54) |
| Finland | -3.77%(-4.27,-3.26) |
| Armenia | -2.73%(-2.93,-2.53) |
| Germany | -2.72%(-3.14,-2.31) |
| Turkmenistan | -2.61%(-3.1,-2.11) |

ASDR: age-standardized DALY rate. EAPC: estimated annual percentage change. CI: confidence interval.The above data has been adjusted by DisMod MR version 2.1.
