# Supplementary material for: Global, regional, and national analyses of the burden of colorectal cancer attributable to diet low in milk from 1990 to 2019: longitudinal observational study
Source: Front Nutr. 2024 Jul 22;11:1431962. doi: 10.3389/fnut.2024.1431962 (PMC11299434; doi:10.3389/fnut.2024.1431962)
Supplement: SUPPLEMENTARY TABLE S7 — Percentage change across countries of the fraction of all colorectal cancer deaths attributable to diet low in milk (95% CI). [file Table_7.docx]

| **Supplementary Table 7.** Percentage change across countries of the fraction of all colorectal cancer deaths attributable to diet low in milk (95% CI). | | | |
| --- | --- | --- | --- |
| **location** | **1990-2010** | **1990-2019** | **2010-2019** |
| Afghanistan | 0.01(-0.01,0.05) | 0.02(-0.01,0.07) | 0.01(0,0.02) |
| Albania | -0.89(-0.96,-0.76) | -0.9(-0.96,-0.76) | -0.08(-0.36,0.22) |
| Algeria | -0.15(-0.36,-0.03) | -0.14(-0.34,-0.03) | 0.01(0,0.04) |
| American Samoa | 0.01(0,0.02) | 0.01(0,0.02) | 0(0,0.01) |
| Andorra | -0.02(-0.15,0.12) | 0(-0.14,0.17) | 0.02(-0.05,0.13) |
| Angola | 0.01(0,0.02) | 0.01(0,0.02) | 0(0,0.01) |
| Antigua and Barbuda | 0.06(0.01,0.15) | 0.06(0.01,0.18) | 0.01(0,0.02) |
| Argentina | 0(-0.01,0.02) | -0.01(-0.05,0) | -0.02(-0.05,0) |
| Armenia | -0.32(-0.66,-0.09) | -0.39(-0.71,-0.12) | -0.1(-0.23,-0.02) |
| Australia | -0.43(-0.68,-0.17) | -0.46(-0.7,-0.21) | -0.04(-0.2,0.12) |
| Austria | -0.17(-0.35,-0.05) | -0.13(-0.3,-0.03) | 0.05(-0.01,0.14) |
| Azerbaijan | -0.03(-0.09,0) | -0.04(-0.13,0) | -0.02(-0.05,0) |
| Bahamas | 0.01(0,0.03) | 0.02(0.01,0.03) | 0(0,0.01) |
| Bahrain | -0.02(-0.05,0) | -0.01(-0.05,0.01) | 0(-0.01,0.01) |
| Bangladesh | -0.01(-0.04,0.01) | -0.01(-0.06,0.01) | 0(-0.01,0.01) |
| Barbados | 0.05(0.01,0.13) | 0.05(0.01,0.13) | 0(0,0) |
| Belarus | 0.81(0.21,2.19) | 0.71(0.19,1.87) | -0.06(-0.15,-0.01) |
| Belgium | -0.21(-0.47,-0.06) | -0.17(-0.4,-0.05) | 0.05(0,0.17) |
| Belize | 0(0,0.01) | 0.01(0,0.01) | 0(0,0) |
| Benin | 0(-0.01,0) | 0(-0.01,0.01) | 0(0,0.01) |
| Bermuda | 0(0,0.01) | 0(0,0.01) | 0(-0.01,0) |
| Bhutan | -0.01(-0.04,0.01) | -0.02(-0.07,0.01) | -0.01(-0.03,0) |
| Bolivia (Plurinational State of) | -0.01(-0.04,0) | -0.01(-0.05,0.01) | 0(-0.01,0) |
| Bosnia and Herzegovina | -0.27(-0.58,-0.07) | -0.27(-0.57,-0.07) | 0(-0.06,0.08) |
| Botswana | 0.01(0,0.02) | 0(-0.02,0.01) | -0.01(-0.04,0) |
| Brazil | -0.12(-0.26,-0.03) | -0.13(-0.27,-0.03) | -0.01(-0.03,0.01) |
| Brunei Darussalam | -0.01(-0.05,0.01) | -0.01(-0.04,0.01) | 0(0,0.01) |
| Bulgaria | 0.06(0.01,0.17) | 0.1(0.02,0.26) | 0.03(0,0.09) |
| Burkina Faso | -0.02(-0.04,0) | -0.02(-0.04,0) | 0(0,0.01) |
| Burundi | 0.01(0,0.02) | 0.01(0,0.02) | 0(0,0) |
| Cabo Verde | -0.04(-0.1,0) | -0.03(-0.1,0) | 0(0,0.01) |
| Cambodia | 0.01(0,0.02) | 0.02(0.01,0.03) | 0.01(0,0.01) |
| Cameroon | 0(-0.01,0.01) | 0(-0.01,0.01) | 0(0,0.01) |
| Canada | -0.28(-0.54,-0.11) | -0.21(-0.43,-0.07) | 0.11(0.03,0.25) |
| Central African Republic | -0.01(-0.02,0) | 0(-0.01,0) | 0.01(0,0.02) |
| Chad | 0(-0.01,0.02) | 0(-0.01,0.03) | 0(-0.01,0.01) |
| Chile | 0.01(0,0.04) | 0.01(0,0.02) | 0(-0.02,0) |
| China | 0.01(-0.01,0.02) | 0.01(-0.01,0.02) | 0(-0.01,0) |
| Colombia | -0.05(-0.15,0) | -0.06(-0.17,0) | -0.01(-0.03,0.01) |
| Comoros | 0.01(0,0.02) | 0(0,0.01) | 0(-0.01,0) |
| Congo | 0.04(0.03,0.06) | 0.04(0.02,0.06) | 0(-0.01,0) |
| Cook Islands | 0(-0.02,0.01) | 0(-0.02,0.01) | 0(-0.01,0) |
| Costa Rica | -0.04(-0.13,0) | -0.02(-0.09,0.02) | 0.02(0,0.09) |
| Côte d'Ivoire | -0.14(-0.33,-0.04) | -0.16(-0.36,-0.05) | -0.02(-0.1,0.03) |
| Croatia | 0.05(0.01,0.15) | 0.05(0.01,0.14) | 0(-0.02,0) |
| Cuba | -0.15(-0.36,-0.02) | -0.11(-0.29,-0.01) | 0.05(0,0.16) |
| Cyprus | 0.1(0.02,0.31) | 0.08(0.01,0.26) | -0.02(-0.06,0) |
| Czechia | -0.12(-0.18,-0.09) | -0.12(-0.18,-0.09) | 0(-0.01,0.01) |
| Democratic People's Republic of Korea | 0(0,0.01) | 0.01(0,0.01) | 0(0,0) |
| Democratic Republic of the Congo | -0.01(-0.02,-0.01) | -0.02(-0.03,-0.01) | 0(-0.01,0) |
| Denmark | -0.26(-0.45,-0.1) | -0.2(-0.39,-0.07) | 0.08(0,0.22) |
| Djibouti | 0(0,0.01) | 0(-0.01,0.01) | 0(-0.01,0.01) |
| Dominica | -0.03(-0.1,0) | -0.02(-0.07,0) | 0.01(0,0.04) |
| Dominican Republic | 0.02(0.01,0.05) | 0.02(0.01,0.05) | 0(-0.01,0) |
| Ecuador | 0.04(0.01,0.13) | 0.05(0.01,0.12) | 0(-0.01,0.01) |
| Egypt | -0.01(-0.05,0.01) | -0.01(-0.05,0.01) | 0(0,0.01) |
| El Salvador | -0.07(-0.21,0) | -0.08(-0.23,0) | 0(-0.02,0) |
| Equatorial Guinea | 0.03(0,0.07) | 0.05(0.02,0.09) | 0.02(0,0.03) |
| Eritrea | 0(-0.01,0) | 0(-0.01,0.01) | 0(0,0.01) |
| Estonia | -0.08(-0.24,-0.01) | -0.11(-0.29,-0.02) | -0.03(-0.09,0.01) |
| Eswatini | 0.04(0,0.13) | 0.04(0,0.12) | 0(-0.02,0) |
| Ethiopia | -0.04(-0.12,0) | -0.05(-0.14,0) | -0.01(-0.03,0) |
| Fiji | 0.03(0.01,0.06) | 0.02(0.01,0.05) | 0(-0.01,0) |
| Finland | -0.43(-0.67,-0.21) | -0.38(-0.64,-0.15) | 0.08(-0.22,0.47) |
| France | -0.05(-0.18,0) | -0.04(-0.16,0.01) | 0.01(-0.02,0.06) |
| Gabon | -0.08(-0.1,-0.06) | -0.07(-0.1,-0.05) | 0(-0.01,0.01) |
| Gambia | 0.01(0,0.02) | 0.01(0,0.02) | 0(0,0.01) |
| Georgia | -0.1(-0.21,0.02) | 0.2(0.07,0.63) | 0.34(0.09,0.99) |
| Germany | -0.23(-0.5,-0.07) | -0.24(-0.52,-0.08) | -0.02(-0.08,0.05) |
| Ghana | 0.05(0.02,0.09) | 0.05(0.02,0.09) | 0(-0.01,0.01) |
| Greece | -0.14(-0.33,-0.03) | -0.1(-0.24,-0.02) | 0.05(0.01,0.15) |
| Greenland | -0.09(-0.23,-0.02) | -0.14(-0.34,-0.04) | -0.05(-0.16,0) |
| Grenada | -0.07(-0.11,-0.05) | -0.08(-0.12,-0.05) | -0.01(-0.02,0) |
| Guam | 0.01(0,0.01) | 0.01(0,0.02) | 0(0,0.01) |
| Guatemala | -0.02(-0.07,0) | -0.02(-0.07,0.01) | 0(0,0.01) |
| Guinea | -0.01(-0.04,0) | -0.02(-0.04,0) | 0(-0.01,0) |
| Guinea-Bissau | 0(-0.01,0) | 0(-0.01,0.01) | 0(0,0.01) |
| Guyana | -0.07(-0.18,0) | -0.07(-0.19,0) | 0(-0.01,0.01) |
| Haiti | 0(-0.01,0.01) | 0(-0.01,0.01) | 0(0,0) |
| Honduras | -0.07(-0.21,0) | -0.06(-0.19,0.01) | 0.01(0,0.04) |
| Hungary | 0.07(0.01,0.2) | 0.05(0,0.17) | -0.02(-0.07,0) |
| Iceland | 1.49(0.4,5.01) | 1.29(0.36,4.39) | -0.08(-0.2,0) |
| India | 0.02(0.01,0.03) | 0.01(0,0.03) | -0.01(-0.02,0) |
| Indonesia | 0(0,0.01) | 0(0,0.01) | 0(0,0) |
| Iran (Islamic Republic of) | -0.05(-0.14,0.01) | -0.04(-0.14,0.01) | 0(0,0.01) |
| Iraq | 0.02(0,0.05) | 0.01(0,0.04) | 0(-0.01,0) |
| Ireland | 1.55(0.5,6.27) | 1.14(0.36,4.63) | -0.16(-0.35,-0.04) |
| Israel | -0.11(-0.27,-0.02) | -0.12(-0.29,-0.02) | -0.01(-0.04,0.01) |
| Italy | -0.16(-0.34,-0.06) | -0.12(-0.27,-0.04) | 0.05(0.01,0.14) |
| Jamaica | -0.06(-0.19,-0.01) | -0.04(-0.13,0) | 0.02(0,0.07) |
| Japan | 0(-0.01,0.01) | 0(-0.01,0.02) | 0(-0.01,0.01) |
| Jordan | -0.05(-0.15,0) | -0.05(-0.14,0) | 0.01(0,0.02) |
| Kazakhstan | -0.69(-0.87,-0.42) | -0.46(-0.72,-0.18) | 0.76(0.35,1.96) |
| Kenya | 0(-0.01,0.02) | 0.05(0.01,0.13) | 0.05(0.01,0.12) |
| Kiribati | -0.01(-0.02,0) | 0(-0.01,0.01) | 0(0,0.01) |
| Kuwait | -0.02(-0.05,0.01) | -0.01(-0.04,0.01) | 0.01(0,0.02) |
| Kyrgyzstan | -0.33(-0.62,-0.13) | -0.29(-0.56,-0.11) | 0.06(-0.08,0.41) |
| Lao People's Democratic Republic | 0.09(0.04,0.14) | 0.09(0.05,0.13) | 0(-0.02,0.01) |
| Latvia | 1.6(0.38,5.96) | 1.41(0.34,5.03) | -0.07(-0.17,-0.01) |
| Lebanon | -0.01(-0.02,0) | -0.01(-0.03,0) | 0(-0.01,0) |
| Lesotho | -0.01(-0.02,0) | -0.01(-0.03,0) | 0(-0.01,0) |
| Liberia | -0.11(-0.15,-0.08) | -0.11(-0.16,-0.08) | -0.01(-0.02,0) |
| Libya | -0.03(-0.08,0) | 0(-0.02,0.01) | 0.03(0.01,0.07) |
| Lithuania | 0.04(0,0.16) | 0.02(-0.03,0.12) | -0.02(-0.08,0.01) |
| Luxembourg | 0.01(-0.17,0.19) | -0.03(-0.24,0.19) | -0.04(-0.16,0.07) |
| Madagascar | 0.04(0,0.12) | 0.05(0.01,0.12) | 0(-0.01,0.01) |
| Malawi | 0(0,0.01) | 0(0,0.01) | 0(0,0) |
| Malaysia | -0.24(-0.33,-0.19) | -0.25(-0.35,-0.2) | -0.01(-0.02,0) |
| Maldives | 0.01(-0.01,0.02) | 0(-0.01,0.02) | 0(-0.01,0) |
| Mali | -0.11(-0.29,-0.02) | -0.09(-0.24,-0.02) | 0.02(0,0.07) |
| Malta | -0.03(-0.09,0) | -0.06(-0.16,-0.01) | -0.03(-0.09,0) |
| Marshall Islands | 0(0,0.01) | 0.01(0,0.02) | 0(0,0.01) |
| Mauritania | 0.11(0.01,0.33) | 0.15(0.03,0.47) | 0.04(0.01,0.12) |
| Mauritius | 0.18(0.13,0.26) | 0.17(0.12,0.25) | 0(-0.01,0.01) |
| Mexico | -0.07(-0.19,-0.01) | -0.07(-0.19,-0.01) | 0(-0.01,0.02) |
| Micronesia (Federated States of) | 0(-0.01,0.01) | 0(0,0.01) | 0(0,0.01) |
| Monaco | -0.11(-0.43,0.22) | -0.31(-0.67,-0.02) | -0.22(-0.49,-0.06) |
| Mongolia | -0.24(-0.53,-0.06) | -0.33(-0.62,-0.11) | -0.12(-0.25,-0.03) |
| Montenegro | -0.34(-0.6,-0.17) | -0.25(-0.51,-0.08) | 0.15(-0.03,0.6) |
| Morocco | -0.07(-0.19,0) | -0.07(-0.2,0) | 0(-0.02,0) |
| Mozambique | 0(0,0) | 0(-0.01,0) | 0(-0.01,0) |
| Myanmar | -0.01(-0.05,0.01) | -0.02(-0.06,0.01) | 0(-0.01,0) |
| Namibia | -0.01(-0.03,0) | -0.02(-0.06,0) | -0.01(-0.03,0) |
| Nauru | 0.01(-0.01,0.03) | 0(-0.01,0.02) | -0.01(-0.02,0.01) |
| Nepal | -0.02(-0.08,0) | -0.03(-0.12,0.01) | -0.01(-0.04,0) |
| Netherlands | 0.33(0.11,0.84) | 0.25(0.07,0.67) | -0.06(-0.16,-0.01) |
| New Zealand | -0.46(-0.61,-0.22) | -0.28(-0.44,-0.11) | 0.34(0.11,0.64) |
| Nicaragua | -0.06(-0.2,0.01) | -0.06(-0.21,0.01) | 0(-0.01,0.01) |
| Niger | -0.02(-0.05,0) | -0.01(-0.04,0) | 0.01(0,0.02) |
| Nigeria | 0(0,0.01) | -0.01(-0.03,0) | -0.01(-0.03,0) |
| Niue | 0(-0.01,0) | 0(-0.01,0) | 0(-0.01,0) |
| North Macedonia | -0.12(-0.29,-0.02) | -0.16(-0.37,-0.03) | -0.04(-0.12,-0.01) |
| Northern Mariana Islands | 0.02(0.01,0.03) | 0.02(0.01,0.03) | 0(0,0) |
| Norway | -0.11(-0.26,-0.02) | -0.12(-0.29,0) | 0(-0.1,0.1) |
| Oman | -0.07(-0.21,-0.01) | -0.06(-0.18,-0.01) | 0.01(0,0.03) |
| Pakistan | -0.1(-0.24,-0.02) | -0.05(-0.13,-0.01) | 0.06(0.01,0.15) |
| Palau | 0(0,0.01) | 0(-0.01,0.01) | 0(0,0) |
| Palestine | 0(0,0.01) | 0(-0.01,0.01) | 0(-0.01,0) |
| Panama | 0.06(0.03,0.11) | 0.05(0.03,0.09) | -0.01(-0.02,0) |
| Papua New Guinea | 0(-0.01,0.01) | 0(0,0.01) | 0(-0.01,0.01) |
| Paraguay | -0.05(-0.16,0) | -0.07(-0.18,-0.01) | -0.01(-0.04,0) |
| Peru | 0.01(0,0.01) | 0.01(0,0.01) | 0(0,0.01) |
| Philippines | -0.22(-0.29,-0.16) | -0.23(-0.31,-0.18) | -0.02(-0.04,-0.01) |
| Poland | 0.23(0.06,0.62) | 0.18(0.05,0.49) | -0.04(-0.1,-0.01) |
| Portugal | -0.39(-0.67,-0.15) | -0.34(-0.61,-0.12) | 0.08(0,0.24) |
| Puerto Rico | -0.01(-0.02,0) | -0.01(-0.02,0) | 0(0,0.01) |
| Qatar | -0.03(-0.09,-0.01) | -0.03(-0.1,0) | 0(-0.02,0.01) |
| Republic of Korea | -0.06(-0.16,0) | -0.05(-0.13,0) | 0.01(0,0.03) |
| Republic of Moldova | 0.15(0.03,0.5) | 0.12(0.02,0.42) | -0.03(-0.08,0) |
| Romania | -0.69(-0.85,-0.41) | -0.37(-0.61,-0.09) | 1(0.46,2.31) |
| Russian Federation | -0.24(-0.42,-0.1) | 0.19(0.04,0.46) | 0.56(0.2,1.18) |
| Rwanda | -0.01(-0.02,0) | -0.01(-0.03,0.01) | 0(-0.01,0.01) |
| Saint Kitts and Nevis | 0(0,0.02) | 0(0,0.02) | 0(-0.01,0) |
| Saint Lucia | -0.05(-0.08,-0.03) | -0.05(-0.09,-0.03) | 0(-0.01,0) |
| Saint Vincent and the Grenadines | -0.01(-0.04,0) | -0.01(-0.04,0) | 0(0,0) |
| Samoa | -0.02(-0.06,0) | -0.02(-0.05,0) | 0(0,0.01) |
| San Marino | -0.13(-0.31,-0.02) | 0(-0.13,0.16) | 0.15(0.04,0.41) |
| Sao Tome and Principe | -0.2(-0.27,-0.15) | -0.23(-0.31,-0.18) | -0.04(-0.07,-0.02) |
| Saudi Arabia | -0.04(-0.13,0) | -0.05(-0.13,0) | 0(-0.01,0) |
| Senegal | 0(0,0.01) | 0(0,0.01) | 0(-0.01,0) |
| Serbia | 0.08(0.02,0.24) | 0.13(0.03,0.44) | 0.05(0.01,0.17) |
| Seychelles | 0.02(0.01,0.05) | 0.02(-0.01,0.04) | -0.01(-0.02,0) |
| Sierra Leone | 0(-0.01,0) | -0.01(-0.01,0) | 0(-0.01,0) |
| Singapore | -0.03(-0.09,0) | -0.04(-0.12,0) | -0.01(-0.04,0) |
| Slovakia | -0.01(-0.04,0) | -0.02(-0.07,0) | -0.01(-0.04,0) |
| Slovenia | -0.14(-0.3,-0.04) | -0.13(-0.3,-0.03) | 0.01(-0.07,0.11) |
| Solomon Islands | 0(-0.01,0.02) | 0(0,0.01) | 0(-0.01,0.01) |
| Somalia | 0(-0.01,0.01) | 0(-0.01,0.01) | 0(-0.01,0) |
| South Africa | -0.03(-0.1,0) | -0.03(-0.11,0) | 0(-0.01,0.01) |
| South Sudan | 0(-0.01,0) | 0(-0.01,0.01) | 0(0,0.01) |
| Spain | 0.03(-0.1,0.24) | 0.15(-0.01,0.53) | 0.12(0.01,0.32) |
| Sri Lanka | 0.02(0.01,0.04) | 0.02(0.01,0.03) | 0(-0.01,0) |
| Sudan | -0.01(-0.04,0) | -0.02(-0.04,0) | 0(-0.01,0) |
| Suriname | 0.03(0,0.09) | 0.03(0.01,0.08) | 0(-0.01,0) |
| Sweden | -0.02(-0.15,0.17) | -0.01(-0.18,0.21) | 0(-0.12,0.14) |
| Switzerland | 0.08(0.01,0.19) | 0.04(-0.04,0.15) | -0.04(-0.12,0.01) |
| Syrian Arab Republic | -0.05(-0.16,0.01) | -0.01(-0.07,0.02) | 0.04(0.01,0.11) |
| Taiwan (Province of China) | -0.03(-0.1,0.01) | -0.03(-0.1,0.01) | 0(-0.01,0) |
| Tajikistan | 0.06(0.01,0.17) | 0.05(0.01,0.13) | -0.01(-0.04,0.01) |
| Thailand | -0.05(-0.1,-0.01) | -0.05(-0.1,-0.01) | 0(0,0) |
| Timor-Leste | 0.05(0.03,0.08) | 0.05(0.02,0.07) | 0(-0.01,0) |
| Togo | 0.01(0,0.02) | 0.01(0,0.03) | 0(0,0.01) |
| Tokelau | 0(-0.01,0) | -0.01(-0.02,0) | 0(-0.01,0) |
| Tonga | 0(-0.01,0) | 0(-0.01,0) | 0(0,0) |
| Trinidad and Tobago | 0.01(0,0.02) | -0.01(-0.03,0) | -0.02(-0.04,-0.01) |
| Tunisia | -0.16(-0.37,-0.03) | -0.16(-0.36,-0.03) | 0.01(-0.01,0.04) |
| Turkey | 0.12(0.04,0.35) | 0.16(0.04,0.49) | 0.03(0,0.11) |
| Turkmenistan | -0.22(-0.45,-0.06) | -0.35(-0.61,-0.13) | -0.17(-0.32,-0.04) |
| Tuvalu | 0(-0.01,0) | 0(-0.01,0) | 0(0,0) |
| Uganda | -0.04(-0.1,0) | -0.04(-0.11,0) | 0(-0.01,0) |
| Ukraine | 0.42(0.1,1.02) | 0.78(0.22,2.59) | 0.25(0.06,0.82) |
| United Arab Emirates | -0.06(-0.16,-0.01) | -0.03(-0.1,0) | 0.03(0.01,0.08) |
| United Kingdom | 0.01(-0.04,0.06) | 0.01(-0.05,0.08) | 0(-0.03,0.04) |
| United Republic of Tanzania | -0.03(-0.08,0) | -0.03(-0.1,0) | 0(-0.02,0) |
| United States of America | -0.11(-0.24,-0.04) | -0.13(-0.27,-0.04) | -0.02(-0.09,0.04) |
| United States Virgin Islands | -0.02(-0.06,0) | -0.01(-0.04,0) | 0.01(0,0.02) |
| Uruguay | 0.05(-0.01,0.17) | -0.1(-0.22,-0.01) | -0.14(-0.29,-0.04) |
| Uzbekistan | 0.18(0.06,0.44) | 0.33(0.09,1.06) | 0.13(0.02,0.46) |
| Vanuatu | 0(-0.01,0.01) | 0.01(0,0.02) | 0(0,0.01) |
| Venezuela (Bolivarian Republic of) | 0(0,0.01) | 0.01(0,0.01) | 0(0,0.01) |
| Viet Nam | -0.01(-0.04,0) | -0.01(-0.04,0) | 0(0,0) |
| Yemen | -0.01(-0.01,0) | 0(-0.01,0) | 0(0,0.01) |
| Zambia | 0.01(0,0.02) | 0.01(0,0.02) | 0(-0.01,0.01) |
| Zimbabwe | 0.02(0,0.05) | 0.02(0,0.05) | 0(-0.01,0) |

CI: confidence interval.The above data has been adjusted by DisMod MR version 2.1.
