# Supplementary material for: Global, regional, and national analyses of the burden of colorectal cancer attributable to diet low in milk from 1990 to 2019: longitudinal observational study
Source: Front Nutr. 2024 Jul 22;11:1431962. doi: 10.3389/fnut.2024.1431962 (PMC11299434; doi:10.3389/fnut.2024.1431962)
Supplement: SUPPLEMENTARY TABLE S8 — Percentage change across countries of the fraction of all colorectal cancer DALYs attributable to diet low in milk (95% CI). [file Table_8.docx]

| **Supplementary Table 8.** Percentage change across countries of the fraction of all colorectal cancer DALYs attributable to diet low in milk (95% CI). | | | |
| --- | --- | --- | --- |
| **location** | **1990-2010** | **1990-2019** | **2010-2019** |
| Albania | -0.9(-0.96,-0.76) | 0.01(-0.01,0.02) | 0(-0.01,0.01) |
| Algeria | -0.14(-0.36,-0.01) | 0(-0.01,0.01) | 0(-0.01,0.01) |
| American Samoa | 0.02(0,0.03) | -0.22(-0.31,-0.17) | -0.02(-0.04,-0.01) |
| Andorra | -0.01(-0.13,0.1) | 0.03(0.01,0.04) | 0.01(0,0.02) |
| Angola | 0(-0.01,0.02) | 0.03(0.01,0.05) | 0(0,0.01) |
| Antigua and Barbuda | 0.06(0.01,0.18) | -0.03(-0.12,0.02) | -0.01(-0.05,0.01) |
| Argentina | 0.01(0,0.02) | 0.09(0.01,0.28) | -0.01(-0.06,0) |
| Armenia | -0.32(-0.65,-0.08) | 0.01(0,0.02) | 0(0,0.01) |
| Australia | -0.48(-0.73,-0.2) | 0.01(-0.01,0.03) | 0(-0.01,0.02) |
| Austria | -0.19(-0.38,-0.05) | -0.27(-0.58,-0.07) | -0.01(-0.07,0.06) |
| Azerbaijan | -0.02(-0.08,0.01) | 0.06(0.02,0.15) | 0(-0.03,0.03) |
| Bahamas | 0.01(0,0.03) | -0.25(-0.35,-0.19) | -0.01(-0.02,0) |
| Bahrain | -0.01(-0.05,0.01) | -0.49(-0.79,-0.19) | 0.86(0.38,2.84) |
| Bangladesh | 0(-0.04,0.03) | 0.03(0.01,0.05) | 0.01(0,0.02) |
| Barbados | 0.06(0.01,0.14) | 1.44(0.34,5.38) | -0.06(-0.15,-0.01) |
| Belarus | 0.8(0.22,2.12) | 0.01(-0.13,0.16) | 0.02(-0.04,0.11) |
| Belgium | -0.23(-0.5,-0.07) | -0.04(-0.12,0) | -0.01(-0.04,0) |
| Belize | 0.01(0,0.02) | 0.01(0,0.02) | 0(0,0.01) |
| Benin | 0(-0.02,0.01) | -0.02(-0.07,0) | -0.01(-0.04,0) |
| Bermuda | 0(0,0.01) | 0.01(-0.01,0.02) | 0.01(0,0.02) |
| Bhutan | 0(-0.04,0.02) | -0.02(-0.09,0.02) | 0(-0.01,0) |
| Bolivia (Plurinational State of) | -0.01(-0.04,0.01) | 0.35(0.11,1.15) | 0.14(0.03,0.46) |
| Bosnia and Herzegovina | -0.26(-0.57,-0.06) | 0.01(-0.03,0.03) | 0.01(0,0.02) |
| Botswana | 0.01(0,0.03) | -0.01(-0.04,0.01) | 0(0,0.01) |
| Brazil | -0.11(-0.26,-0.02) | -0.04(-0.09,0) | 0(0,0.01) |
| Brunei Darussalam | 0(-0.03,0.02) | -0.39(-0.71,-0.12) | -0.11(-0.24,-0.02) |
| Bulgaria | 0.05(0.01,0.15) | -0.9(-0.97,-0.77) | -0.05(-0.25,0.28) |
| Burkina Faso | -0.02(-0.05,0) | 0.13(0.03,0.4) | -0.02(-0.08,0) |
| Burundi | 0(-0.01,0.02) | 0.04(0.02,0.06) | 0(-0.01,0) |
| Cabo Verde | -0.04(-0.11,0.01) | -0.16(-0.36,-0.05) | -0.03(-0.11,0.02) |
| Cambodia | 0.01(0,0.03) | -0.31(-0.62,-0.09) | -0.11(-0.24,-0.02) |
| Cameroon | -0.01(-0.02,0.01) | -0.43(-0.71,-0.12) | 1.17(0.48,3.55) |
| Canada | -0.31(-0.6,-0.12) | -0.5(-0.75,-0.24) | -0.04(-0.19,0.14) |
| Central African Republic | -0.01(-0.02,0) | 0.7(0.2,1.86) | -0.06(-0.15,0) |
| Chad | 0(-0.02,0.02) | -0.01(-0.05,0.02) | 0(-0.01,0.01) |
| Chile | 0.02(0,0.04) | 0(-0.02,0.02) | 0.01(-0.01,0.03) |
| China | 0.03(0.01,0.04) | 0.01(-0.01,0.03) | 0(-0.01,0.01) |
| Colombia | -0.04(-0.14,0.01) | 0.76(0.21,2.61) | 0.25(0.06,0.82) |
| Comoros | 0.02(0,0.03) | 0.18(0.05,0.56) | 0.34(0.09,1.07) |
| Congo | 0.04(0.02,0.07) | -0.46(-0.69,-0.27) | 0.12(-0.11,0.59) |
| Cook Islands | 0.01(-0.01,0.02) | 0.01(0,0.02) | 0.01(-0.01,0.02) |
| Costa Rica | -0.05(-0.15,0) | 0.18(0.05,0.5) | -0.04(-0.1,-0.01) |
| Croatia | -0.14(-0.33,-0.04) | 0.04(0.02,0.05) | 0(-0.01,0) |
| Cuba | 0.06(0.02,0.17) | -0.04(-0.13,0.02) | 0.01(0,0.03) |
| Cyprus | -0.17(-0.41,-0.02) | -0.15(-0.37,-0.02) | -0.04(-0.12,-0.01) |
| Czechia | 0.11(0.02,0.32) | 0.09(0.05,0.14) | 0.01(-0.01,0.02) |
| Côte d'Ivoire | -0.12(-0.18,-0.09) | -0.36(-0.65,-0.13) | 0.08(0,0.24) |
| Democratic People's Republic of Korea | 0.01(0,0.02) | -0.11(-0.29,-0.02) | -0.03(-0.09,0.01) |
| Democratic Republic of the Congo | -0.01(-0.03,0) | 0.04(0.01,0.15) | -0.02(-0.07,0) |
| Denmark | -0.29(-0.52,-0.12) | -0.27(-0.53,-0.1) | 0.15(-0.02,0.6) |
| Djibouti | 0.01(0,0.02) | 0.09(0.01,0.24) | 0.03(0,0.09) |
| Dominica | -0.03(-0.1,0) | -0.13(-0.28,-0.04) | 0.05(0.01,0.14) |
| Dominican Republic | 0.04(0.02,0.07) | -0.01(-0.05,0.01) | 0(0,0.01) |
| Ecuador | 0.05(0.01,0.13) | -0.29(-0.56,-0.1) | 0.06(-0.08,0.38) |
| Egypt | 0(-0.04,0.03) | -0.23(-0.48,-0.07) | 0.12(0.03,0.3) |
| El Salvador | -0.07(-0.22,0.01) | 0(-0.01,0.01) | 0(-0.01,0.01) |
| Equatorial Guinea | 0.02(-0.01,0.06) | -0.25(-0.53,-0.08) | -0.02(-0.08,0.04) |
| Eritrea | 0(-0.02,0.01) | -0.23(-0.44,-0.08) | 0.1(0.01,0.26) |
| Estonia | -0.08(-0.24,-0.01) | -0.02(-0.17,0.17) | 0.02(-0.08,0.16) |
| Eswatini | 0.04(0,0.13) | -0.06(-0.18,0) | 0.01(-0.03,0.05) |
| Ethiopia | -0.05(-0.12,0) | 0.01(-0.05,0.07) | 0(-0.03,0.03) |
| Fiji | 0.04(0.01,0.07) | -0.05(-0.09,-0.03) | 0(0,0.01) |
| Finland | -0.51(-0.75,-0.32) | 0.04(0.02,0.07) | 0(-0.01,0.01) |
| France | -0.06(-0.19,-0.01) | -0.06(-0.16,-0.01) | -0.03(-0.09,0) |
| Gabon | -0.08(-0.1,-0.06) | -0.13(-0.3,-0.03) | 0.01(-0.06,0.1) |
| Gambia | 0.01(0,0.02) | 0.19(0.05,0.45) | 0.59(0.21,1.27) |
| Georgia | -0.12(-0.27,-0.02) | 0.01(0.01,0.03) | 0(-0.01,0) |
| Germany | -0.24(-0.52,-0.07) | -0.32(-0.59,-0.1) | -0.15(-0.3,-0.03) |
| Ghana | 0.05(0.01,0.1) | -0.35(-0.55,-0.15) | 0.47(0.14,0.94) |
| Greece | -0.15(-0.35,-0.03) | -0.19(-0.44,-0.05) | 0.05(0,0.16) |
| Greenland | -0.09(-0.23,-0.01) | -0.12(-0.29,-0.02) | -0.01(-0.04,0.01) |
| Grenada | -0.08(-0.12,-0.05) | 0.14(0.03,0.46) | 0.05(0.01,0.17) |
| Guam | 0.02(0,0.03) | 0(-0.04,0.02) | 0(-0.01,0.01) |
| Guatemala | -0.02(-0.07,0.01) | -0.04(-0.17,0.04) | 0.02(0,0.05) |
| Guinea | -0.02(-0.05,0) | 1.44(0.4,5.14) | -0.07(-0.18,-0.01) |
| Guinea-Bissau | -0.01(-0.02,0.01) | -0.02(-0.13,0.03) | 0.01(0,0.02) |
| Guyana | -0.06(-0.18,0) | -0.01(-0.04,0) | -0.02(-0.05,0) |
| Haiti | 0(-0.01,0.02) | 0.13(-0.02,0.48) | 0.12(0.01,0.34) |
| Honduras | -0.05(-0.2,0.02) | 0.02(0.01,0.04) | 0.01(0,0.01) |
| Hungary | 0.07(0.01,0.19) | -0.02(-0.07,0) | 0.02(0,0.05) |
| Iceland | 1.63(0.43,6.02) | 0.02(0.01,0.03) | 0(0,0.01) |
| India | 0.03(0.02,0.04) | 0.02(-0.03,0.1) | -0.02(-0.07,0.01) |
| Indonesia | 0.01(0,0.02) | 0.01(-0.02,0.02) | 0.03(0.01,0.08) |
| Iran (Islamic Republic of) | -0.03(-0.13,0.02) | 0.07(0.01,0.21) | 0.01(0,0.03) |
| Iraq | 0.02(-0.01,0.05) | -0.06(-0.19,0.01) | -0.01(-0.04,0.01) |
| Ireland | 1.59(0.51,6.28) | 0.02(0.01,0.03) | 0(0,0.01) |
| Israel | -0.11(-0.27,-0.02) | -0.14(-0.31,-0.03) | 0(-0.09,0.1) |
| Italy | -0.18(-0.36,-0.06) | -0.11(-0.27,-0.02) | 0.05(0.01,0.14) |
| Jamaica | -0.07(-0.19,-0.01) | 0.04(-0.03,0.13) | -0.04(-0.12,0) |
| Japan | 0(-0.01,0.01) | -0.06(-0.19,0) | 0(-0.01,0.01) |
| Jordan | -0.05(-0.15,0.01) | 0(-0.02,0.01) | 0.01(0,0.01) |
| Kazakhstan | -0.73(-0.92,-0.45) | 0.04(0.01,0.1) | 0(0,0.01) |
| Kenya | 0.01(-0.01,0.03) | -0.05(-0.15,0.01) | 0(-0.03,0.01) |
| Kiribati | -0.01(-0.03,0.01) | 0.01(-0.03,0.04) | 0.01(0,0.02) |
| Kuwait | 0(-0.04,0.02) | 0.28(0.09,0.7) | -0.06(-0.15,-0.01) |
| Kyrgyzstan | -0.33(-0.61,-0.12) | -0.12(-0.34,-0.01) | 0.02(0,0.04) |
| Lao People's Democratic Republic | 0.09(0.04,0.14) | -0.04(-0.13,0) | 0.02(0,0.07) |
| Latvia | 1.61(0.38,6.08) | -0.05(-0.25,0.14) | -0.03(-0.15,0.07) |
| Lebanon | 0(-0.02,0.01) | -0.08(-0.13,-0.05) | -0.01(-0.01,0) |
| Lesotho | 0(-0.02,0.01) | -0.05(-0.18,0.01) | 0.01(0,0.02) |
| Liberia | -0.11(-0.16,-0.08) | -0.04(-0.13,0.01) | 0.01(0,0.02) |
| Libya | -0.02(-0.08,0.01) | -0.15(-0.33,-0.04) | 0.05(-0.01,0.16) |
| Lithuania | 0.04(0,0.14) | 0.06(0.02,0.14) | 0.01(-0.01,0.02) |
| Luxembourg | -0.02(-0.19,0.14) | -0.12(-0.27,-0.02) | 0(-0.03,0.02) |
| Madagascar | 0.05(0.01,0.14) | -0.07(-0.18,-0.01) | -0.14(-0.29,-0.04) |
| Malawi | 0(-0.01,0.02) | -0.01(-0.03,0.01) | 0(-0.01,0) |
| Malaysia | -0.24(-0.33,-0.19) | -0.02(-0.1,0.01) | 0.03(0,0.1) |
| Maldives | 0.01(-0.01,0.02) | 0.06(0.02,0.16) | 0(-0.02,0) |
| Mali | -0.12(-0.3,-0.02) | -0.01(-0.06,0.02) | 0(-0.03,0.01) |
| Malta | -0.03(-0.09,0) | -0.15(-0.36,-0.02) | 0.01(-0.01,0.04) |
| Marshall Islands | 0.02(0,0.04) | 0(-0.04,0.04) | 0(-0.01,0.02) |
| Mauritania | 0.11(0.01,0.34) | 0(-0.02,0.01) | -0.02(-0.03,0) |
| Mauritius | 0.19(0.14,0.27) | 0(-0.01,0.02) | 0(-0.01,0.01) |
| Mexico | -0.06(-0.19,0.01) | -0.12(-0.32,-0.02) | 0.06(0.01,0.18) |
| Micronesia (Federated States of) | 0(-0.01,0.02) | 0(-0.02,0.01) | 0(-0.01,0.01) |
| Monaco | -0.11(-0.4,0.23) | -0.13(-0.25,-0.04) | -0.02(-0.09,0.04) |
| Mongolia | -0.23(-0.52,-0.05) | -0.03(-0.11,0.02) | -0.01(-0.04,0.01) |
| Montenegro | -0.36(-0.62,-0.17) | 0.06(0.02,0.14) | 0.05(0.01,0.13) |
| Morocco | -0.06(-0.19,0) | 0.05(0.01,0.09) | 0.02(0,0.04) |
| Mozambique | 0(-0.01,0.01) | -0.07(-0.22,0.01) | 0(-0.02,0.01) |
| Myanmar | -0.01(-0.05,0.02) | 0.06(0.03,0.1) | -0.01(-0.02,0) |
| Namibia | -0.01(-0.04,0.01) | -0.03(-0.09,0) | 0(-0.01,0.01) |
| Nauru | 0.01(-0.02,0.03) | 0.01(0,0.03) | 0.01(-0.01,0.02) |
| Nepal | -0.02(-0.07,0.01) | 0.01(-0.02,0.06) | 0.01(-0.01,0.03) |
| Netherlands | 0.36(0.11,0.93) | 1.19(0.38,4.49) | -0.15(-0.35,-0.04) |
| New Zealand | -0.55(-0.74,-0.28) | -0.03(-0.1,0.01) | 0(-0.01,0.01) |
| Nicaragua | -0.05(-0.18,0.03) | -0.03(-0.12,0.02) | 0(-0.01,0.01) |
| Niger | -0.02(-0.05,0.01) | 0.02(-0.05,0.06) | 0.06(0.03,0.12) |
| Nigeria | 0(-0.01,0.01) | -0.01(-0.02,0.01) | 0(-0.01,0.01) |
| Niue | 0(-0.01,0.01) | -0.04(-0.19,0.04) | 0(-0.01,0.01) |
| North Macedonia | -0.12(-0.29,-0.01) | 0.06(0.02,0.14) | 0(0,0.01) |
| Northern Mariana Islands | 0.02(0.01,0.04) | -0.06(-0.19,0.01) | 0(-0.02,0.01) |
| Norway | -0.14(-0.28,-0.05) | 0.01(-0.02,0.02) | -0.01(-0.03,0.01) |
| Oman | -0.07(-0.2,0) | 0(-0.01,0.01) | 0.01(0,0.02) |
| Pakistan | -0.11(-0.24,-0.03) | -0.05(-0.14,0) | 0(-0.02,0.01) |
| Palau | 0.01(0,0.01) | 0.02(0,0.05) | 0(-0.01,0.01) |
| Palestine | 0.01(0,0.03) | 0.01(0,0.03) | 0.01(-0.01,0.02) |
| Panama | 0.07(0.04,0.12) | 0(-0.04,0.02) | 0.01(0,0.02) |
| Papua New Guinea | 0(-0.02,0.03) | -0.01(-0.03,0.01) | 0(0,0.01) |
| Paraguay | -0.05(-0.16,0) | 0.02(0.01,0.04) | 0(-0.02,0.01) |
| Peru | 0.02(0.01,0.03) | -0.01(-0.07,0.02) | 0.01(0,0.02) |
| Philippines | -0.21(-0.28,-0.15) | 0(-0.02,0.02) | 0.01(0,0.02) |
| Poland | 0.23(0.06,0.65) | -0.06(-0.18,0) | 0.01(0,0.03) |
| Portugal | -0.41(-0.7,-0.16) | 0.02(0.01,0.03) | 0.01(0,0.01) |
| Puerto Rico | 0(-0.02,0) | -0.02(-0.03,0) | 0(-0.01,0.01) |
| Qatar | -0.03(-0.09,0) | 0.01(0,0.03) | 0(-0.01,0.01) |
| Republic of Korea | -0.05(-0.15,0.02) | 0.01(-0.01,0.02) | 0(-0.01,0.01) |
| Republic of Moldova | 0.15(0.03,0.48) | 0.01(-0.01,0.03) | -0.01(-0.02,0) |
| Romania | -0.74(-0.91,-0.44) | 0(-0.01,0) | 0(-0.01,0.01) |
| Russian Federation | -0.25(-0.44,-0.1) | -0.01(-0.06,0.01) | 0(-0.02,0.01) |
| Rwanda | -0.01(-0.02,0.01) | -0.07(-0.1,-0.05) | 0(-0.01,0.02) |
| Saint Kitts and Nevis | 0(-0.01,0.01) | -0.05(-0.11,-0.01) | 0(-0.01,0.01) |
| Saint Lucia | -0.05(-0.09,-0.03) | -0.03(-0.05,-0.01) | 0(-0.01,0.01) |
| Saint Vincent and the Grenadines | -0.01(-0.03,0.01) | -0.02(-0.09,0.02) | 0.01(0,0.03) |
| Samoa | -0.01(-0.05,0.01) | -0.01(-0.04,0.01) | 0(-0.01,0.02) |
| San Marino | -0.12(-0.3,-0.02) | 0.02(0,0.04) | 0(-0.01,0.01) |
| Sao Tome and Principe | -0.21(-0.28,-0.15) | -0.03(-0.1,0.01) | 0(-0.02,0.01) |
| Saudi Arabia | -0.04(-0.12,0.01) | 0(-0.01,0.02) | 0(-0.01,0.01) |
| Senegal | 0(-0.01,0.02) | 0.02(0.01,0.04) | 0(-0.01,0.01) |
| Serbia | 0.08(0.02,0.26) | 0(0,0.01) | 0(-0.01,0) |
| Seychelles | 0.03(0.01,0.05) | 0(-0.02,0.01) | 0(-0.01,0.02) |
| Sierra Leone | -0.01(-0.02,0) | -0.1(-0.24,-0.02) | 0.02(0,0.07) |
| Singapore | -0.03(-0.08,0) | 0.02(0,0.05) | -0.01(-0.02,0) |
| Slovakia | -0.01(-0.04,0) | 0(-0.02,0.01) | 0(-0.01,0.01) |
| Slovenia | -0.14(-0.3,-0.04) | -0.02(-0.09,0.01) | 0.04(0.02,0.08) |
| Solomon Islands | 0.01(-0.01,0.03) | -0.01(-0.03,0.01) | 0(-0.01,0.01) |
| Somalia | -0.01(-0.02,0.01) | 0.04(0.01,0.13) | 0(-0.01,0.01) |
| South Africa | -0.03(-0.1,0.01) | 0(-0.02,0.02) | 0(-0.02,0.01) |
| South Sudan | 0(-0.02,0.01) | 0.16(0.05,0.49) | 0.03(0,0.12) |
| Spain | 0(-0.13,0.18) | 0.06(0.01,0.14) | 0(-0.01,0.01) |
| Sri Lanka | 0.04(0.02,0.06) | 0(-0.01,0.01) | 0(0,0.01) |
| Sudan | -0.02(-0.04,0) | -0.06(-0.13,-0.02) | 0.06(0.01,0.16) |
| Suriname | 0.04(0.01,0.1) | 0.02(0,0.03) | 0(0,0.01) |
| Sweden | -0.04(-0.17,0.12) | 0(-0.02,0.01) | 0(-0.01,0.01) |
| Switzerland | 0.09(0.03,0.18) | 0(-0.02,0) | 0(0,0.01) |
| Syrian Arab Republic | -0.03(-0.15,0.03) | -0.24(-0.32,-0.18) | -0.04(-0.07,-0.02) |
| Taiwan (Province of China) | -0.02(-0.09,0.02) | -0.01(-0.03,0) | -0.01(-0.03,0) |
| Tajikistan | 0.06(0.01,0.19) | 0.01(0,0.03) | 0(-0.01,0.02) |
| Thailand | -0.04(-0.1,0) | 0(-0.01,0.02) | 0(-0.01,0.01) |
| Timor-Leste | 0.06(0.03,0.09) | -0.14(-0.33,-0.03) | -0.05(-0.16,0) |
| Togo | 0.02(0,0.04) | -0.01(-0.03,0.02) | 0(-0.01,0.01) |
| Tokelau | 0(-0.01,0.01) | -0.02(-0.04,0) | 0(-0.01,0.01) |
| Tonga | 0(-0.01,0.01) | 0(-0.02,0.01) | 0.01(-0.01,0.02) |
| Trinidad and Tobago | 0.01(0.01,0.02) | 0.01(0,0.02) | 0(0,0.01) |
| Tunisia | -0.16(-0.37,-0.02) | 0.02(0.01,0.04) | 0(-0.01,0.01) |
| Turkey | 0.13(0.04,0.34) | 0.01(0,0.02) | 0.01(0,0.01) |
| Turkmenistan | -0.2(-0.43,-0.05) | -0.3(-0.64,-0.03) | -0.22(-0.48,-0.07) |
| Tuvalu | 0(-0.01,0.01) | 0(-0.01,0.01) | 0(-0.01,0) |
| Uganda | -0.05(-0.11,-0.01) | -0.02(-0.05,0) | 0(-0.01,0.01) |
| Ukraine | 0.4(0.1,1.04) | 0.02(0.01,0.04) | 0.01(0,0.01) |
| United Arab Emirates | -0.06(-0.16,-0.01) | 0.01(-0.01,0.02) | 0(-0.01,0.01) |
| United Kingdom | 0(-0.04,0.06) | 0.04(0.03,0.07) | 0(-0.01,0.01) |
| United Republic of Tanzania | -0.03(-0.08,0.01) | 0(-0.01,0.01) | 0(-0.01,0.01) |
| United States of America | -0.11(-0.22,-0.04) | 0(-0.12,0.13) | 0.14(0.04,0.38) |
| United States Virgin Islands | -0.01(-0.05,0.01) | -0.12(-0.17,-0.08) | 0(-0.01,0.02) |
| Uruguay | 0.08(0.02,0.22) | -0.12(-0.16,-0.08) | 0(-0.02,0.01) |
| Uzbekistan | 0.19(0.06,0.48) | 0.19(0.14,0.27) | 0(-0.01,0.01) |
| Vanuatu | 0(-0.01,0.02) | 0.05(0.01,0.1) | 0(-0.02,0.03) |
| Venezuela (Bolivarian Republic of) | 0.01(0,0.02) | 0.15(0.02,0.48) | 0.04(0.01,0.12) |
| Viet Nam | -0.01(-0.04,0.01) | -0.01(-0.02,0) | 0(-0.01,0.01) |
| Yemen | -0.01(-0.02,0) | 0.02(0.01,0.04) | 0(-0.01,0.01) |
| Zambia | 0.01(-0.01,0.03) | 0(-0.03,0.01) | 0.01(0,0.02) |
| Zimbabwe | 0.02(0,0.06) | 0(-0.01,0.01) | 0(-0.01,0.01) |

DALYs: disability-adjusted life-years. CI: confidence interval.The above data has been adjusted by DisMod MR version 2.1.
